# The effect of age on the intestinal mucus thickness, microbiota composition and immunity in relation to sex in mice

**DOI:** 10.1371/journal.pone.0184274

**Published:** 2017-09-12

**Authors:** Marlies Elderman, Bruno Sovran, Floor Hugenholtz, Katrine Graversen, Myrte Huijskes, Eva Houtsma, Clara Belzer, Mark Boekschoten, Paul de Vos, Jan Dekker, Jerry Wells, Marijke Faas

**Affiliations:** 1 Top Institute Food and Nutrition, Wageningen, the Netherlands; 2 Division of Medical Biology, department of Pathology and Medical Biology, University of Groningen, Groningen, the Netherlands; 3 Host-Microbe Interactomics Group, Wageningen University, Wageningen, the Netherlands; 4 Laboratory of Microbiology, Wageningen University and Research, Wageningen, the Netherlands; 5 Nutrition, Metabolism and Genomics group, Wageningen University, Wageningen, the Netherlands; 6 Department of Obstetrics and Gynaecology, University of Groningen and University Medical Centre Groningen, Groningen, the Netherlands; Mayo Clinic Rochester, UNITED STATES

## Abstract

A mucus layer covers and protects the intestinal epithelial cells from direct contact with microbes. This mucus layer not only prevents inflammation but also plays an essential role in microbiota colonization, indicating the complex interplay between mucus composition-microbiota and intestinal health. However, it is unknown whether the mucus layer is influenced by age or sex and whether this contributes to reported differences in intestinal diseases in males and females or with ageing. Therefore, in this study we investigated the effect of age on mucus thickness, intestinal microbiota composition and immune composition in relation to sex. The ageing induced shrinkage of the colonic mucus layer was associated with bacterial penetration and direct contact of bacteria with the epithelium in both sexes. Additionally, several genes involved in the biosynthesis of mucus were downregulated in old mice, especially in males, and this was accompanied by a decrease in abundances of various *Lactobacillus* species and unclassified *Clostridiales type IV* and *XIV* and increase in abundance of the potential pathobiont *Bacteroides vulgatus*. The changes in mucus and microbiota in old mice were associated with enhanced activation of the immune system as illustrated by a higher percentage of effector T cells in old mice. Our data contribute to a better understanding of the interplay between mucus-microbiota-and immune responses and ultimately may lead to more tailored design of strategies to modulate mucus production in targeted groups.

## 1. Introduction

The human gut harbours trillions of microbes, which are called the microbiota. These microbes are fermenters of non-digestible food components and modulators of the immune system. They are of crucial importance for the regulation and maintenance of health [1,2]. The microbiota are separated from the host inner milieu by the so-called intestinal barrier. This barrier is responsible for the protection of the host against invaders such as pathogens and is under the control of several immunological and non-immunological components. Any disturbance in this barrier function can lead to increased gut permeability, or the so-called “leaky gut” [3]. This dysfunction of the gut barrier has been suggested to contribute to a broad range of disorders including food allergy, intestinal bowel disease (IBD), cancer, and even auto-immune diseases such as Diabetes type 1 [4]. Both age and sex are known to influence the pathophysiology of intestinal diseases [5,6], but the mechanisms underlying these differences are not known yet.

During the last decade the intestinal barrier has been extensively studied [7–10], especially with respect to the permeability of the intestinal epithelial layer. Recently, it has been shown that the mucus layer contributes to maintenance of barrier function [11,12]. The intestinal mucus layer is a viscoelastic gel located on top of the epithelial cells. The mucus is synthesized by the goblet cells, located in between the intestinal enterocytes [13]. In the large intestine two separate mucus layers can be distinguished by histological methods [14–16]. The so-called firm inner layer that is largely free of microbiota and the loose layer that serves not only as barrier but also as substrate for mucus consuming organisms. MUC2 is the main colonic secreted mucin and is the major component of both the inner and outer mucus layers [14]. This mucus prevents contact between luminal bacteria and the epithelium and thereby prevents inflammation [14].

Our recent studies have demonstrated that mice lacking *Muc2* (*Muc2*^-/-^) or producing less mucus (*Muc2*^+/-^) have an altered composition, diversity and richness of the microbiota in both the ileum and colon [12,17]. *Muc2*^-/-^ mice develop colitis around four weeks of age and the abundance of *Bacteroides* pathobionts increased before histological signs of pathology [10]. The production of a thinner mucus layer in heterozygous *Muc2* ^+/-^ mice results in more frequent contact of the epithelium with luminal bacteria, differential expression of genes participating in mucosal stress responses and exacerbation of a transient inflammatory state around the time of weaning [12,17,18]. These studies demonstrate an essential interplay between mucus composition, microbiota and intestinal health. Many questions, however, are still unanswered with respect to mucus and intestinal health. It is not known whether mucus thickness is influenced by age, and whether the process of ageing is sex specific. Also it is not known whether the mucus integrity is involved in the reported gradual deterioration of barrier function in elderly [19].

To gain a better understanding of the influence of age on the dynamic interplay between mucus-microbiota-immune responses and intestinal barrier function, we investigated mucus thickness, intestinal microbiota composition and immune composition in young (3 months) and old (19 months) C57B1/6OlaHsd (B6) mice. To study whether potential differences between young and old mice are sex dependent, we used both male and female mice. Additionally, we investigated these parameters in a group of females in which ovariectomy was performed at 15 months to mimic the effects of menopause in human females [20]. The results may ultimately lead to more tailored design of strategies to modulate mucus production in targeted groups.

## 2. Methods

### 2.1. Mice

Male and female C57B1/6OlaHsd (B6) mice were purchased from Harlan (Harlan, Horst, The Netherlands) at an age of eight weeks. Mice were co-housed (5 mice per cage, according to sex) in isolated ventilated cages to limit environmental influences. The mice had *ad libitum* access to food (D12450B diet; Research Diets Services, Wijk bij Duurstede, the Netherlands) and water. The mice were sacrificed at an age of 3 (n = 10) and 19 months (n = 10) by cervical dislocation under anesthesia (isoflurane and oxygen). Subsequently their Peyer’s patches (PP) and spleens were removed for immune cell analysis. Colonic tissues were fixed in Carnoy’s fixative and embedded in paraffin for study of the mucus. For microarray analysis approximately 1 cm of distal ileum and proximal colon was frozen in liquid nitrogen and stored at -80°C prior to RNA isolation and transcriptomics. For microbiota analysis feces from the last part of the colon were collected for MITChip analysis [21]. Additionally, feces of the old mice (19 months) were collected at 8, 13, and 15 months. All young female mice were sacrificed during the diestrus phase of their ovarian cycle to ensure low stable levels of progesterone and estrogens. In addition, we included a group of female mice (n = 10) that were ovariectomized (ovx) at 15 months of age to determine the effect of a loss of female sex hormones (human menopause) [20]. These mice were also sacrificed at the age of 19 months. During the ovariectomy procedure, the mice were anesthetized with isoflurane and oxygen and two small incisions were made on both ventral sides. Both ovaries were localized, ligated and removed. After surgery the mice received analgesic (Temgesic; 10 μl/g body weight). All mice experiments were performed after receiving approval of the Animal Care Committee of the Groningen University. S1 Table shows the weight of the mice.

### 2.2. Bacterial DNA extraction and microbiota profiling

Total DNA was extracted from the feces samples (n = 5 per group) using the repeated bead-beating-plus column method [22]. The microbiota composition was determined using the Mouse Intestinal Tract Chip (MITChip), a diagnostic 16S rRNA array, which consists of 3,580 unique probes designed to profile murine intestinal microbiota [21]. Briefly, for MITChip, 16S rRNA gene amplification of the bacterial DNA, *in vitro* transcription, labeling, and hybridization were carried out as described previously [23]. Data were normalized and analyzed using a set of R-based scripts in combination with a custom-designed relational database, which operates under the MySQL database management system. For microbial profiling, the Robust Probabilistic Averaging (RPA) signal intensities of 2667 specific probes for the 94 genus-level bacterial groups detected on the MITChip, were used [24]. Diversity calculations were performed using a microbiome R-script package (https://github.com/microbiome). The Redundancy analysis (RDA) was performed in Canoco 5.0, where variables were tested for their significance by the Monte Carlo permutation, and visualized in triplots [25].

### 2.3. Histology

Paraffin sections (5 μm) of the colon were cut using a microtome Microm HM350, (Thermo scientific, Germany) and attached to poly-L-lysine-coated glass slides (Thermo scientific, Germany). After overnight incubation at 37°C, slides were deparaffinised and hydrated step-wise using 100% xylene followed by several solutions of distilled water containing decreasing amounts of ethanol. Sections were stained with hematoxylin and eosin (H&E) and PAS/Alcian blue.[26] Mucus layer thickness was measured (10 measurements per section / 2 sections per mouse / 5 mice per condition) using Image J software (NIH, Maryland, USA).

### 2.4. Fluorescent in situ hybridization (FISH) and MUC2-staining

Paraffin sections (5 μm) were deparaffinised with xylene and a series of ethanol solutions to 100% ethanol. The tissue sections were incubated with the universal bacterial probe EUB338 (5’-GCTGCCTCCCGTAGGAGT-3’) (Isogen Bioscience BV, De Meern, the Netherlands) conjugated to Alexa Fluor488. A ‘non-sense’ probe (5’-CGACGGAGGGCATCCTCA-3’) conjugated to Cy3, was used as a negative control. Tissue sections were incubated overnight with 0.5 μg of probe in 50 μL of hybridization solution (20 mmol/L Tris-HCl (pH 7.4), 0.9 mol/L NaCl, 0.1% (w/v) SDS) at 50°C in a humid environment using a coverslip to prevent drying of the sample. The sections were washed with (20 mmol/L Tris-HCl (pH 7.4), 0.9 mol/L NaCl) at 50°C for 20 min and then washed 3 times in PBS for 10 min in the dark and incubated with DRAQ5 (Invitrogen) (1:1000) for 1 h at 4°C to stain nuclei. Sections were washed 3 times in PBS for 10 min, mounted in fluoromount G (SouthernBiotec, Alabama, USA) and stored at 4°C. For the MUC2 staining, paraffin sections (5 μm) were deparaffinised and rehydrated and antigen retrieval was performed by heating the sections for 20 min in 0.01 M sodium citrate (pH 6.0) at 100°C. Sections were washed for 3 h with 3 changes of Tris-Buffered Saline (TBS). Non-specific binding was reduced using 10% (*v/v*) goat serum (Invitrogen, Life technologies Ltd, Paisley, UK) in TBS for 30 min at room temperature. MUC2 was detected by staining the sections with anti-MUC2 antibody (kindly gifted by Dr. Gunnar Hansson, Gothenburg University, Sweden) diluted 1:500 in TBS, and goat-anti-rabbit Alexa 488 conjugated antibody (1:1000) (Molecular Probes, Life Technologies Ltd, Paisley, UK) in TBS.

### 2.5. Intestinal microarray analysis

For microarray analysis, RNA was purified from the distal ileum and proximal colon of mice (n = 5 per group) using TRIzol (Life Technologies, Calsbad, CA, USA) followed by an additional round of purification with RNeasy Minikit columns (Qiagen, Venlo, the Netherlands). The quality of RNA was determined using RNA 6000 nanochips on the Agilent 2100 bioanalyzer (Agilent Technologies, Amsterdam, the Netherlands). Purified RNA (100 ng) was labeled with the Affymetrix WT PLUS reagent kit (Affymetrix, Santa Clara, CA, USA) and hybridized to an Affymetrix Mouse Gene 1.1 ST array plate (Affymetrix, Santa Clara, CA, USA). Hybridization, washing and scanning were carried out on an Affymetrix GeneTitan platform according to the manufacturer's instructions. Arrays were normalized using the robust multi-array average method [27,28]. Probe sets were defined according to Dai *et al*. (2005) [29]. In this method probes are assigned to Entrez IDs as a unique gene identifier. The *p*-values were calculated using an intensity-based moderated t-statistic (IBMT) [30]. Only probe sets with a fold-change of at least 1.2 (up/down) and a q-value (corrected p-value) < 0.05 were considered to be significantly different.

To gain insight into the biological role of the genes which were differently expressed between young and old mice and males and females, we analyzed the differentially expressed genes in the ileum and colon of young (3 months) and old (19 months) male and female mice using Ingenuity Pathway Analysis (IPA) (Ingenuity System). IPA uses a comprehensive expert-curated repository of biological interactions and functional annotations, mainly acquired from peer-reviewed scientific publications that provide the building blocks for network construction. IPA annotations follow the gene ontology (GO) annotation principle, but are based on a patented knowledge base of > 1,000,000 protein-protein interactions. GO annotations are used by ingenuity in order to investigate, among others, overrepresented biological functions. The IPA output includes biological functions and signaling pathways with statistical assessment of the significance of their representation based on Fisher’s Exact Test. Here, this test calculates the probability that genes participate in a given biological function relative to their occurrence in all other biological function annotations. The input was all the differentially regulated genes (p-value < 0.05, fold-change > 1.2) in the ileum and colon.

### 2.6. Isolation of spleen and Peyer’s patches cells

Single cell suspensions of spleens and PP were made by mechanical disruption of the tissues between two object glasses in 2 ml ice cold RPMI containing 10% heat inactivated fetal calf serum (FCS). Splenic red blood cells were eliminated by incubation with 4 ml ice-cold ammonium chloride. Falcon tubes with cell strainer caps (Corning, Amsterdam, the Netherlands) (35 μm) were used to remove cell clumps before the cells were counted and used for staining.

### 2.7. Cell staining

Spleen and PP cells were stained for T and B cell populations and dendritic cells (DCs). T cells were determined using CD3, and further subdivided into T cytotoxic (CD8^+^) and T helper (CD4^+^) cells. Within the CD8^+^ and CD4^+^ cell subsets, expression of CD69, CD62L and CD44 were measured. In another panel we determined the expression of T helper cell subsets using the markers CD3, CD8, Tbet (Th1), Gata3 (Th2), RORɣt (Th17) and FoxP3^+^CD25^+^ (Treg). B cells were determined using CD19 and B220. Within the B cells the expression of IgA was determined. Within the DCs (MHC2^+^CD64^-^CD19^-^CD11c^+^) expression of CD103 was assessed. Antibody specifications are described in S2 Table. All antibodies were diluted in a volume of 25 μl, supplemented to a volume of 25 μl with FACS buffer (PBS + 10% FCS (*v/v*)). Approximately 0.1x10^6^ spleen or PP cells for the T and B cell panels and 0.4x10^6^ spleen or PP cells for the Th cell and DC panel were incubated for 20 minutes in FACS buffer (10% FCS (*v/v*)) containing 20% (*v/v*) normal rat serum (Jackson, Newmarket, UK), and for the DC panel also 2% (*v/v*) Fc block (CD16/32) (Biolegend, Uithoorn, the Netherlands), to prevent non-specific antibody binding. This was followed by incubation in an extracellular antibody mix for 30 minutes. Next, the cells were fixed in FACS lysing solution (BD Biosciences, Breda, the Netherlands) for 30 minutes. The B and Th subset samples underwent intracellular staining and were washed twice with a permeabilization buffer (eBioscience, Vienna, Austria) after which they were incubated with an intracellular blocking medium (20% (v/v) rat serum in permeabilization buffer) for 20 minutes. Next, these cells were incubated with an intracellular antibody mix for 30 minutes. Washing was performed in between all incubation steps. The whole procedure was performed on ice and in the dark. Isotype control antibodies were used at the same concentration and purchased from the same company as the extracellular and intracellular antibodies.

### 2.8. Flow cytometry

Cell samples were analyzed using the FACSverse Flow Cytometer system (BD Biosciences, Breda, the Netherlands), using FACSsuite software. Analysis was performed using FlowJo version 10 software (FlowJo, LLC, Oregon, USA). Two panels were made to select T cells (see also gating strategy in S1 and S2 Figs). In the first panel, lymphocytes were gated based on size in the forward side scatter plot and T cells were determined by selecting CD3^+^ cells. Within the CD3^+^ cells, CD4^+^ and CD8^+^ cells were selected. Within both the CD4^+^ and CD8^+^ population, the percentage of cells expressing CD69, CD62L and CD44 was measured. CD62L^+^CD44^-^ are indicated as naïve cells, CD62L^+^CD44^+^ are indicated as central memory (CM) cells and CD62L^-^CD44^+^ are indicated as effector memory (EM) cells. In the second T cell panel, within the CD4^+^ cells, the percentage of cells expressing Tbet, Gata3, RORɣt, FoxP3 and CD25 was assessed. B cells were determined as CD19^+^ and B220^+^ cells. Within the B cells the expression of IgA was determined. To gate DCs, first living cells were selected based on size in the forward side scatter plot. Next MHC2^+^ and CD64^-^ cells were selected to exclude macrophages. Within the MHC2^+^CD64^-^ population, first B cells were excluded with CD19 and subsequently DCs were selected as CD11c^+^. Within the DCs the expression of CD103 was measured (S3 Fig). Isotype controls were set at 1% and are shown in S1–S3 Figs.

### 2.9. Statistics

Data for mucus thickness are expressed as the mean with standard error of the mean (SEM). For analyzing significant effects of age and sex on the thickness of the mucus layer a Mann-Whitney U test was used. Microbiota Shannon diversity and richness data and flow cytometry data are expressed as dot plots + means. The overall effect of age and sex was determined with a Two-way ANOVA (TWA), followed by a Bonferroni post-test when an interaction between age and sex was found. P-values of 0.05 or smaller were considered statistically significant and p-values between 0.05 and 0.1 were defined as a trend.

## 3. Results

### 3.1 Aging induced decline in mucus thickness in the colon is not influenced by sex

**Fig 1:**
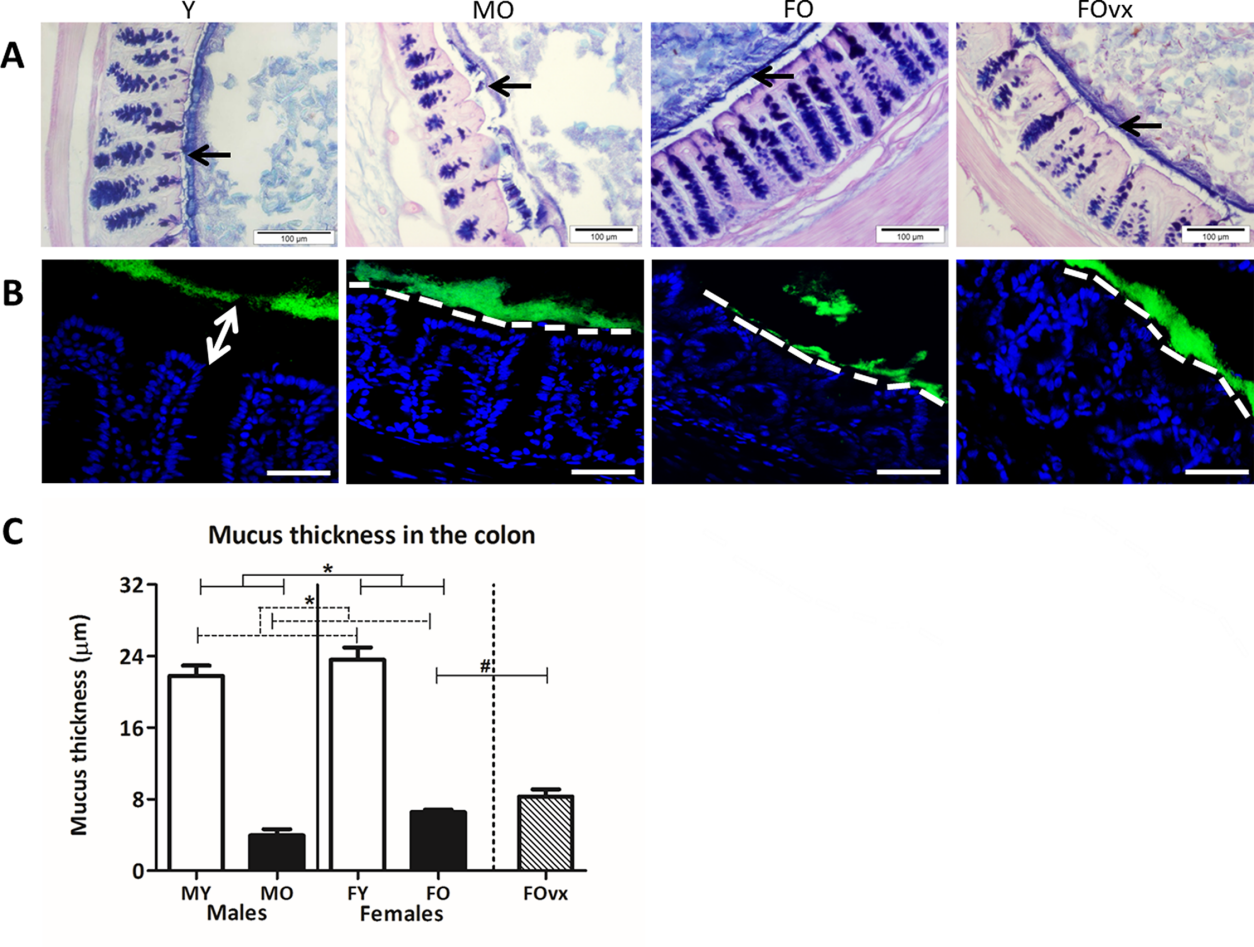
The effect of age and sex on the mucus thickness in the colon. Representative pictures of PAS/Alcian Blue staining of the colon of young (Y) (3 months), old male (MO) (19 months), old female (FO) (19 months) and old ovariectomized female (FOvx) (19 months) B6 mice. Since young male and female mice showed a similar mucus layer morphology, only one representative image is shown for young mice. The mucus is indicated in blue and black arrows. Scale bars: 100 μm (A). Representative pictures of FISH staining of the colon of young, old male, old female and old ovx female mice, using the general bacterial probe EUB338-Alexa Fluor 488 (green), and nuclear staining DRAQ5 (blue). The apical membranes of the epithelial cells are indicated by a dashed white line. Arrow represents the gap between bacteria and epithelium in young healthy colon. This gap is not observed in colon from old mice, and the bacteria are close to the epithelium. Scale bars: 50 μm (B). Mucus thickness measured in PAS/Alcian Blue stained sections (10 measurements per section and 2 sections per mouse) in 5 colonic tissues of young and old mice. Significant effects are indicated with an asterisk (*) (Mann-Whitney U test, p<0.05). An additional group of ovariectomized (ovx) old females was added and compared with the old females to determine the effect of a loss of female sex hormones (human menopause) (C).

To study the effect of ageing and sex on mucus thickness a PAS/Alcian Blue staining (Fig 1) and MUC2 immunofluorescent staining (S4 Fig) was performed on colon tissue samples fixed in Carnoy. We analyzed the data with a TWA, to evaluate if there was an interaction between age and sex on the mucus thickness in the colon. When and interaction was present we tested with a Bonferroni post-test whether the effect of age was the same in both sexes. Overall, in old mice the firm mucus layer was thinner (<10 μm) than in young mice (20–25 μm) and females had a thicker mucus layer than males (TWA, p<0.05) (Fig 1A and 1C). However, the effect of age on the mucus thickness was not different between males and females, neither did ovariectomy changed the mucus thickness (Fig 1C). FISH staining was performed to study interaction of bacteria and epithelial cells. As shown in Fig 1B, the colonic mucus layer of old males and females (both non-ovx and ovx) mice demonstrated many locations where the mucus failed to prevent intestinal microbiota from contact with the epithelium.

### 3.2. Ageing induced changes in intestinal microbiota composition, diversity, and richness is influenced by sex

**Fig 2:**
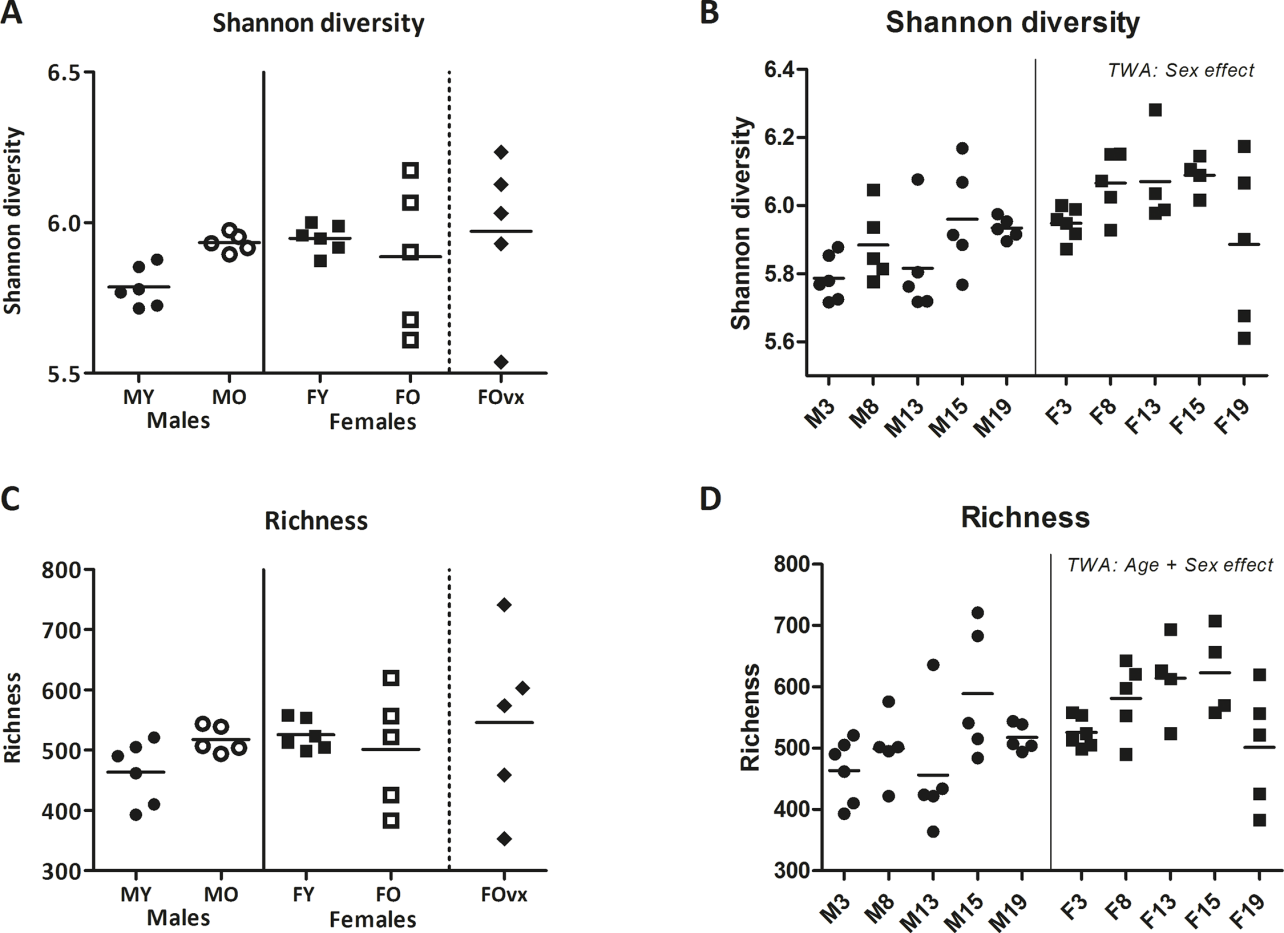
Effect of age and sex on fecal microbiota composition. Graphs showing the Shannon diversity (A) and the richness (C) in the fecal microbiota of male and female and young (3 months) and old (19 months) B6 mice. In addition, we collected feces from the old mice at three previous ages (8, 13 and 15 months). The Shannon diversity (B) and richness (D) of these time points, including the data of the young mice (3 months) are shown. Results are expressed as dot plots + means and were tested using a Two-way ANOVA for overall age and sex effects, followed by a Bonferroni post-test for comparison between groups. An additional group of ovariectomized (ovx) old females was added and compared with the old females to determine the effect of a loss of female sex hormones (human menopause). Significant effects are indicated with an asterisk (*) (p<0.05), while a trend (0.1<p>0.05) is indicated with a hash (#).

As mucus is a substrate for mucolytic bacteria and abnormalities in mucus function are associated with enhanced colonization by pathobionts [12], we determined the microbial composition in the feces of young and old, male and female mice. Additionally, we determined the richness (number of unique species) and Shannon diversity (calculation between richness and evenness (abundance over species)) of the microbiota composition. Overall, there was a tendency for age and sex to interact with each other concerning microbiota diversity (TWA, p = 0.06). However, no significant effect of ageing in both males and females was found (Fig 2A). Age and sex had no effect on the microbiota richness (TWA). Ovariectomy also had no effect on the microbiota diversity or richness (Fig 2A and 2C). From the old mice (19 months) feces were collected at three previous time points (8, 13 and 15 months). Data are shown in Fig 2B and 2D. Overall, age had no effect on the diversity of the microbiota composition, while sex did (TWA, p<0.05), while both sex and age influenced the microbiota richness (TWA, p<0.05).

**Fig 3:**
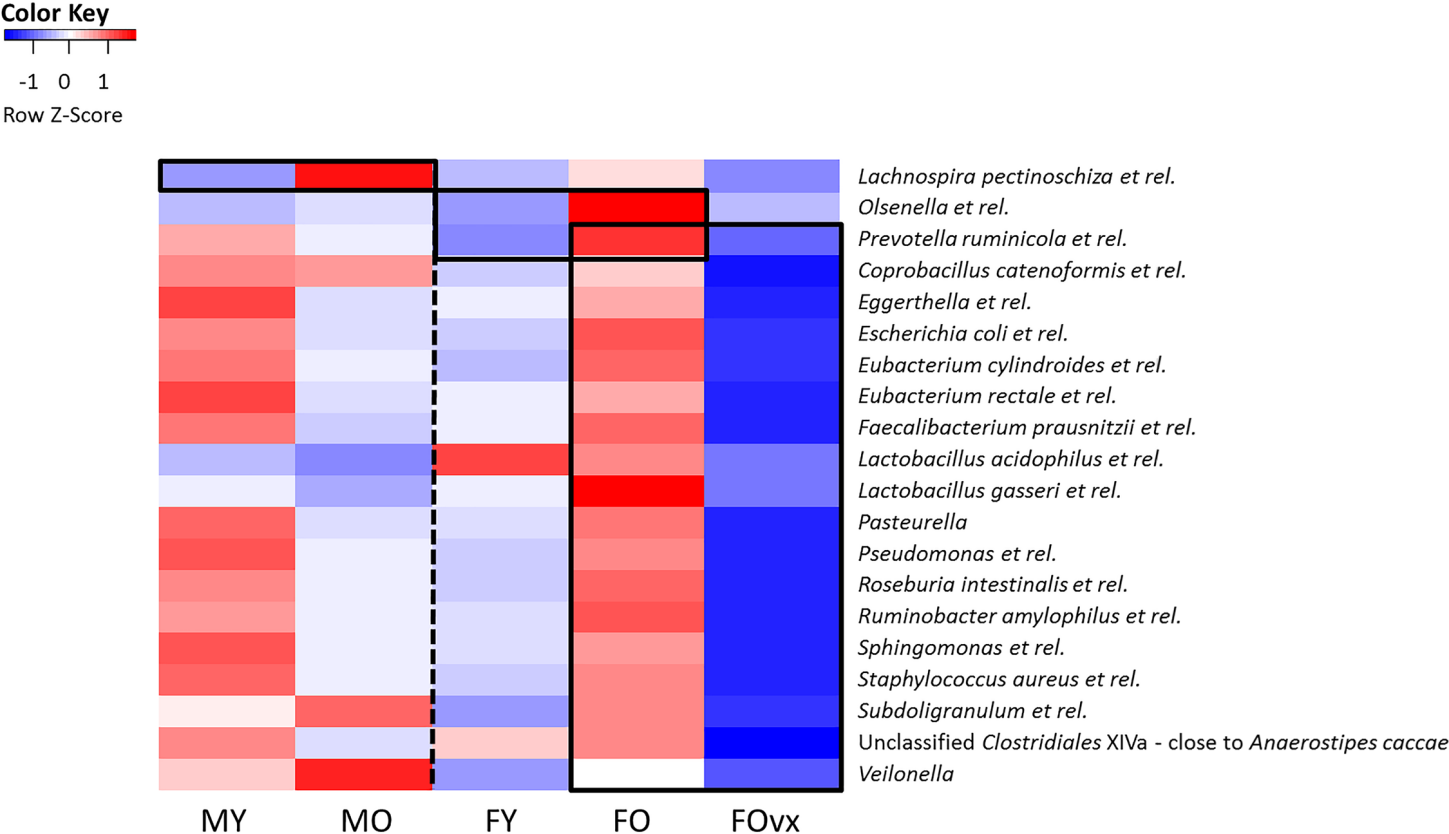
The effect of age on the abundance of several bacteria groups is sex specific. Heat-maps showing the abundance of several bacteria that differed significantly between young (3 months) and old (19 months) males and females. A box indicates bacteria which have a significantly different abundance between the two groups within that box. Colors indicate relative abundances normalized per bacterial group (per row), dark blue is the lowest abundance and dark red the highest abundance detected over all the samples of a bacterial group.

When looking at bacterial groups, we found that overall, aged mice had a higher abundance of *Bacteroides vulgatus et rel*., *Clostridium lactifermentans et rel*., and *uncultured Clostridiales*, while they had a lower abundance of for instance several *Lactobacilli* species and *Unclassified Clostridium IV and XIVa* as compared to young mice (Mann-Whitney U test, p<0.05) (S5 Fig). When looking at the effect of age in males and females separately, we found that aged male mice had a higher abundance of *Lachnospira pectinoschiza et rel*. as compared to young male mice, while old females had a higher abundance of *Olsenella et rel*. and *Prevotella ruminicola et rel*. as compared to young females (Mann-Whitney U test, p<0.05) (Fig 3). Ovariectomy affected the microbiota composition with several microbiota being less abundant in old ovx females than in old non-ovx females, including *Roseburia intestinalis et rel*., *Faecalibacterium prausnitzii et rel*, and *Lactobacillus gasseri et rel*. (Mann-Whitney U test, p<0.05) (Fig 3).

### 3.3. Ageing induced changes in the expression of genes involved in mucus biosynthesis and immune signaling in the ileum and colon is influenced by sex

To determine whether the variation in microbiota composition and mucus thickness between young and old mice and males and females had any effect on gene expression in the intestine, we performed a microarray analysis on tissue from the distal ileum and proximal colon. To better understand the age dependent decrease in mucus thickness, we focused on a number of genes involved in the gut barrier, such as mucus biosynthesis, tight junctions and anti-microbial peptides (S3 and S4 Tables).

In the ileum, none of the *Muc* genes had a different expression between young and old mice, in both sexes. When looking at genes involved in mucus biosynthesis, none of the genes different between young and old males, while old females had a lower expression of *Chst8* and *Tff3* than young females. The effect of age on the expression of genes related to tight junctions was also more pronounced in females than in males. The expression of *Cldn2 and Cldn3* was lower, while the expression of *Ctnna2*, *Ctnna3* (p = 0.06) *Jam2*, *Jam3* and *Mpdz* was higher in old females as compared to young females. None of the genes involved in the synthesis of anti-microbial peptides were affected by age. Ovariectomy had no effect on the expression of genes related to gut barrier in the ileum (S3 Table).

In the colon, *Muc1* expression was significantly lower in the colon of old males than young males. In old females expression of *Muc6* tended (p = 0.07) to be higher than in young females. When looking at genes involved in mucus biosynthesis, the effect of ageing was more pronounced in males. When comparing old males with young males, several genes in the mucus biosynthesis had a lower expression, such as *Chst2 (p = 0*.*09)*, *Chst4* and *St3gal4*, while *St3gal5* tended (p = 0.08) to have a higher expression. From genes related to tight junctions, *Cldn10* tended (p = 0.05) to have a higher expression in old males than young males. In old females, *Cldn11* and *Cldn4* had a higher expression than in young females. Genes involved in the synthesis of anti-microbial peptides, such as *Reg4* and several defensins, were generally expressed at higher levels in old mice as compared to young mice. Ovariectomy also had no effect on the expression of genes related to gut barrier in the colon (S4 Table).

An impaired mucus layer and altered microbiota composition will lead to changes in intestinal immune responses. Therefore, ingenuity pathway analysis (IPA) was applied to identify the biological functions, with focus on immunology, of the genes that differed significantly between young and old mice and between males and females. We took for IPA the z-scores of functions as this is a quantitative measure for up- or downregulation, in which a negative z-score implies an inhibition of the pathway and a positive z-score an enhanced activity.

In the ileum, in both males and females, IPA listed 500 functions that were enriched from the genes which differed in expression between young and old mice, including several immune related functions. However, during ageing many of these functions were influenced in a sex dependent way (S5 Table). Most functions were downregulated in old males as compared to young males. Key immunological functions that were strongly downregulated in old males as compared to young males were the quantity and migration of lymphocytes. In females, less immunological functions related to the genes with a different expression during aging were affected. Key immunological functions that were affected by age in females were adhesion of immune cells and inflammatory response.

In the colon, key immunological functions that were strongly upregulated in old males as compared to young males were differentiation of leukocytes and quantity of neutrophils. On the other hand, activation of B lymphocytes, functions of leukocytes and phagocytes and the quantity of lymphocytes were downregulated in old males as compared to young males. In old females, key immunological functions that were upregulated as compared to young females were activation of antigen presenting cells and phagocytes and inflammatory responses, while the quantity of lymphocytes and the recruitment of granulocytes was downregulated in old females as compared to young females (S6 Table).

### 3.4. Ageing induced changes in T cell development in the Peyer’s patches and spleen is influenced by sex

As we found age and sex differences in mucus, microbiota, and intestinal immune functions using a microarray, we subsequently studied immune cell subsets in the Peyer’s patches (PP) and the spleens. We used the PP since it is an important immune sampling site of antigens from the gut lumen [31]. The spleen was used as a reference for systemic immunity. We focused on T cell subsets, as many genes which were influenced by ageing were related to T cell functions. Again we analyzed the data with a TWA, to understand if there was an interaction between age and sex on the percentage of the immune populations. When and interaction was present we tested with a Bonferroni post-test whether the effect of age was the same in both sexes.

**Fig 4:**
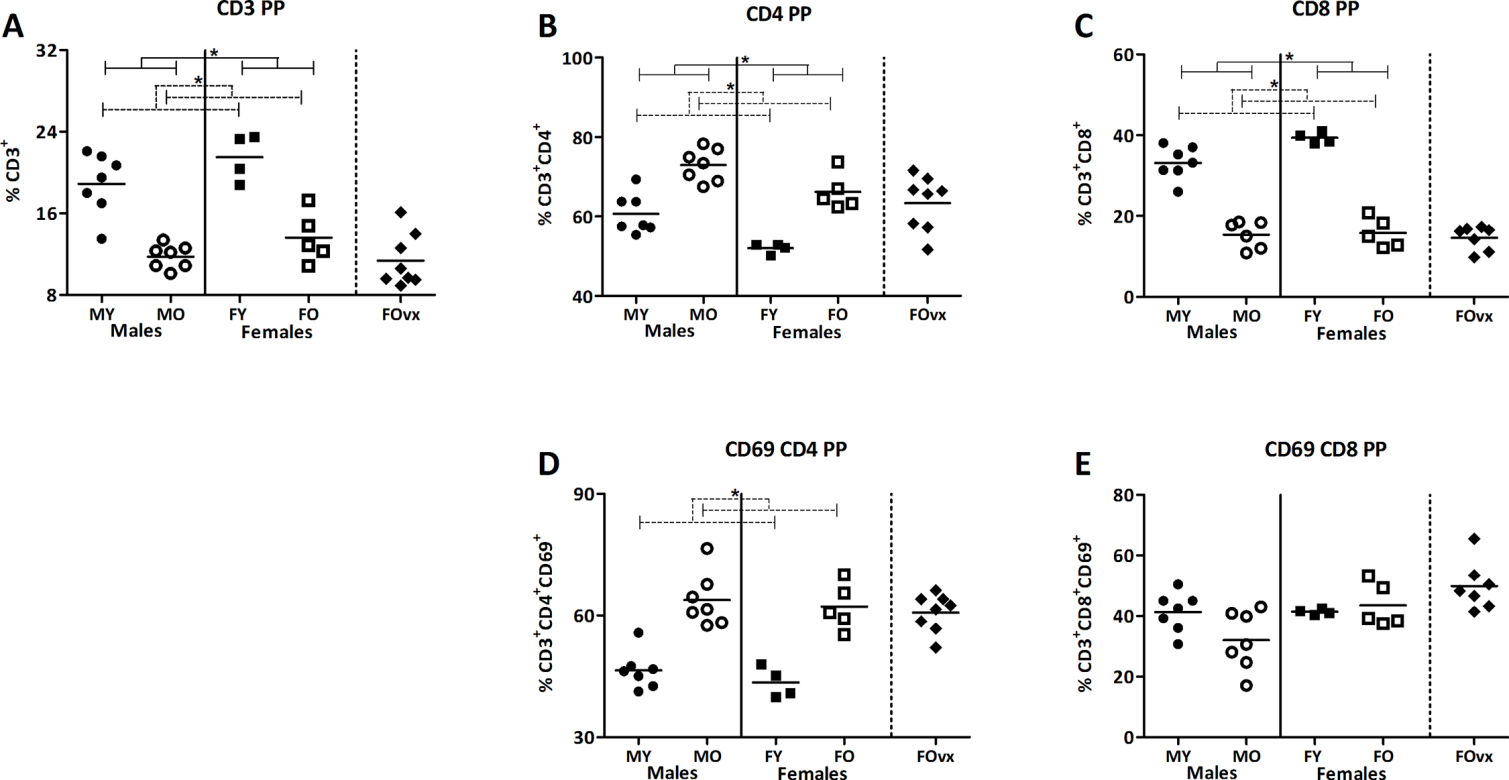
Effect of age and sex on T lymphocytes in the Peyer’s patches. Percentage of CD3^+^ T lymphocytes (A), the percentage of T helper cells (CD4^+^) (B), T cytotoxic cells (CD8^+^) (C), and the percentage of expression of CD69^+^CD4^+^ (D) and CD69^+^CD8^+^ (E) in the Peyer’s patches (PP) of young (3 months) and old (19 months) male and female B6 mice. T helper and T cytotoxic cells are expressed as the frequency of CD4^+^ and CD8^+^ cells within the CD3^+^ population respectively. Results are expressed as dot plots + means and were tested using a Two-way ANOVA for overall age and sex effects, followed by a Bonferroni post-test for comparison between groups. Significant age effects are indicated with dashed lines and significant sex effects are indicated with solid lines (p<0.05). An additional group of ovariectomized (ovx) old females was added and compared with the old females with a t-test to determine the effect of a loss of female sex hormones (human menopause).

In the PP, old mice had a lower percentage of T cells (CD3^+^) than young mice, while males had a higher percentage of T cells than females (TWA, p<0.05), illustrating baseline age and sex differences in T cell percentages (Fig 4A). The percentage of T helper cells (CD4^+^ cells) was overall higher in old mice than in young mice, but was also influenced by sex, as it was overall higher in males than in females (TWA, p<0.05) (Fig 4B). Overall, age and sex also influenced the percentage of T cytotoxic cells (CD8^+^ cells), which was lower in old mice than young mice and lower in males than in females (TWA, p<0.05) (Fig 4C). Age had an overall effect on the percentage of CD4^+^ cells expressing the early activation marker CD69, but not on the percentage of CD8^+^ cells expressing CD69. Old mice had a higher percentage of CD69^+^ CD4^+^ cells than young mice (TWA, p<0.05). Sex had no effect on the percentage of CD4^+^ or CD8^+^ expressing CD69 (Fig 4D and 4E). Ovariectomy, to mimic menopause, had no effect on the percentage of T cells, T cells subsets (CD4^+^ and CD8^+^ cells) and the activation of these cells (CD69^+^ cells) (Fig 4A–4E).

**Fig 5:**
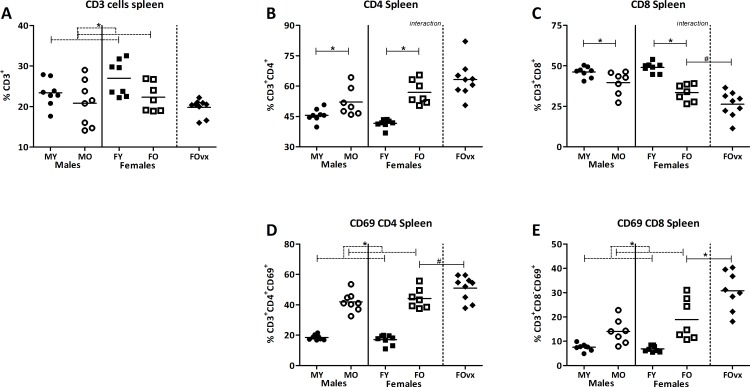
Effect of age and sex on T lymphocytes in the spleen. Percentage of CD3^+^ T lymphocytes (A), the percentage of T helper cells (CD4^+^) (B), T cytotoxic cells (CD8^+^) (C), and the percentage of expression of CD69^+^CD4^+^ (D) and CD69^+^CD8^+^ (E) in the spleen of young (3 months) and old (19 months) male and female B6 mice. T helper and T cytotoxic cells are expressed as the frequency of CD4^+^ and CD8^+^ cells within the CD3^+^ population respectively. Results are expressed as dot plots + means and were tested using Two-way ANOVA, followed by a Bonferroni post-test for comparison between groups. Significant age effects are indicated with dashed lines and significant sex effects are indicated with solid lines (p<0.05). An additional group of ovariectomized (ovx) old females was added and compared with the old females with a t-test to determine the effect of a loss of female sex hormones (human menopause).

Similar observations were seen in the spleen. Old mice had a lower percentage of T cells (CD3^+^) than young mice, while sex had no effect on the percentage of T cells (TWA, p<0.05) (Fig 5A). However, in both the percentage of CD4^+^ and CD8^+^ cells, the effect of age was different in the two sexes (TWA, p<0.05). Old mice had a higher percentage of CD4^+^, while a lower percentage of CD8^+^ cells, but this ageing effect was more pronounced in females (Bonferroni, p<0.05) (Fig 5A and 5C). Additionally, old mice had a higher percentage of both CD4^+^ and CD8^+^ cells expressing CD69, while sex had no effect on these populations (TWA, p<0.05) (Fig 5D and 5E). Ovariectomized females had lower percentage of CD8^+^ cells and both CD4^+^ and CD8^+^ cells expressing CD69 (t-test, p<0.05) (Fig 5A–5E).

**Fig 6:**
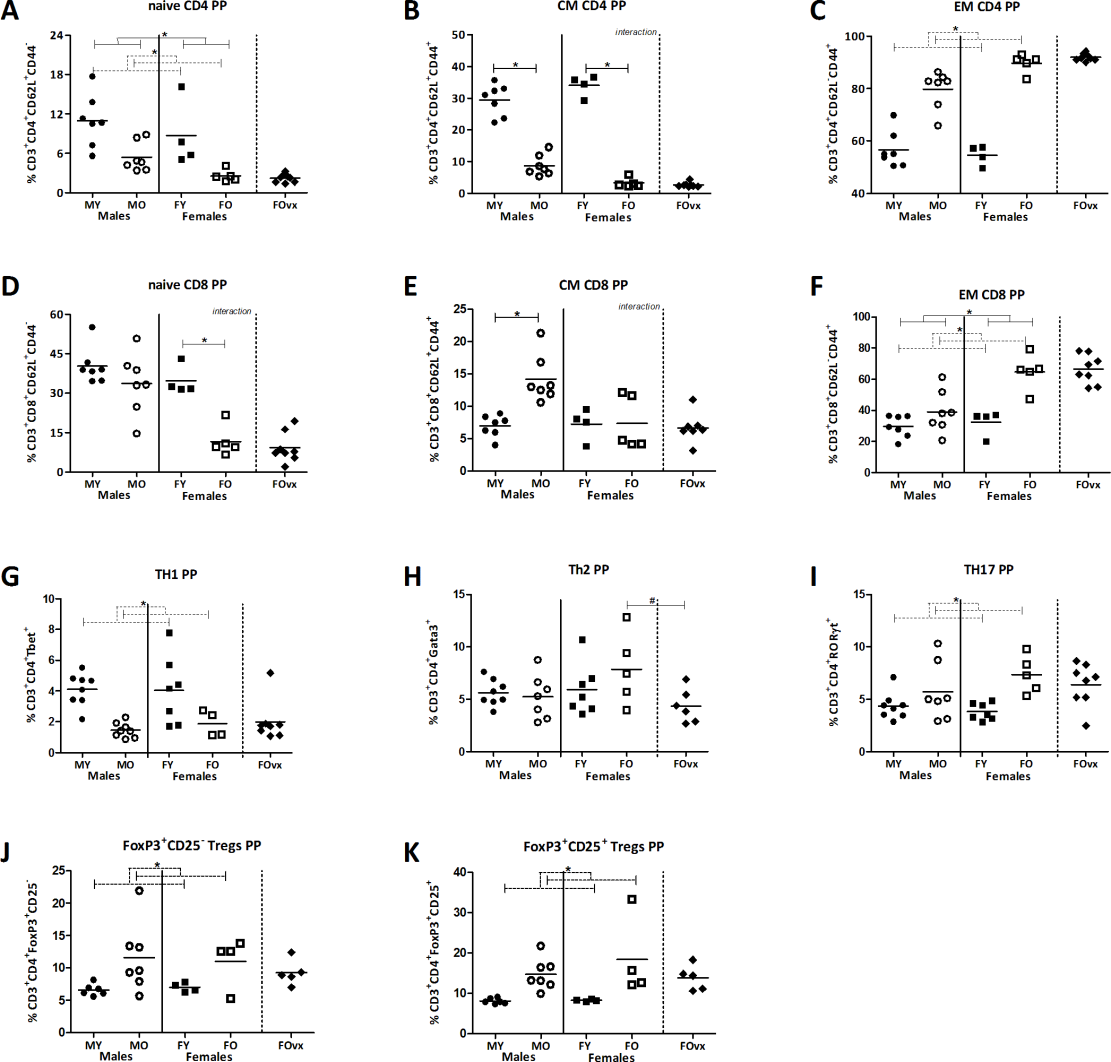
Effect of age and sex on T cell differentiation in the Peyer’s patches. Percentage of CD62L^+^CD44^-^ naive CD4^+^ (A) and CD8^+^ (D), CD62L^+^CD44^+^ central memory CD4^+^ (B) and CD8^+^ (E), CD62L^-^CD44^+^ effector memory CD4^+^ (C) and CD8^+^ (F), T helper 1 (G), T helper 2 (H), T helper 17 (I), FoxP3^+^CD25^-^ T regulatory cells (J), and FoxP3^+^CD25^+^ T regulatory cells (K) in the Peyer’s patches (PP) of young (3 months) and old (19 months) male and female B6 mice. T helper and T cytotoxic cells are expressed as the frequency of CD4^+^ and CD8^+^ cells within the CD3^+^ population respectively. Results are expressed as dot plots + means and were tested using Two-way ANOVA, followed by a Bonferroni post-test for comparison between groups. Significant age effects are indicated with dashed lines and significant sex effects are indicated with solid lines (p<0.05). An additional group of ovariectomized (ovx) old females was added and compared with the old females with a t-test to determine the effect of a loss of female sex hormones (human menopause).

When looking at the maturation status of the T cells in the PP, we found that, old mice had a lower percentage of naïve CD4^+^ cells than young mice, while males had a higher percentage of naïve CD4^+^ cells than females (TWA, p<0.05). However, in the naïve CD8^+^ population, the effect of age was different in males and females (TWA, p<0.05). No effect of age was found in male mice, while old females had a significantly lower percentage of naïve CD8^+^ cells than young females (Bonferroni, p<0.05) (Fig 6A and 6D). The effect of age was also different in both the percentage of central memory (CM) CD4^+^ and CD8^+^ cells (TWA, p<0.05). Both old male and female mice had a lower percentage of CM CD4^+^ cells than young male and female mice respectively, but this effect of age was more pronounced in females (Bonferroni, p<0.05). Age only had a significant effect on the percentage of CM CD8^+^ cells in male mice; old males had a higher percentage than young males (Bonferroni, p<0.05) (Fig 6B and 6E). Furthermore, old mice had a higher percentage of both effector memory (EM) CD4^+^ and CD8^+^ cells than young mice (TWA, p<0.05). Sex only influenced the percentage of EM CD8^+^ cells; males had a lower percentage than females (TWA, p<0.05) (Fig 6C and 6F). Ovariectomy had no effect on the maturation of T cells (Fig 6A–6F).

Age also influenced the percentage of T helper subsets in the PP, while sex did not influence the effect of ageing. Old mice had a lower percentage of Th1 cells than young mice (Fig 6G), while old mice had a higher percentage of Th17 cells, FoxP3^+^CD25^-^ and FoxP3^+^CD25^+^ Tregs than young mice (TWA, p<0.05) (Fig 6I, 6L, and 6F). The effect of ovariectomy was only present in the Th2 subset; old ovx females had a reduced percentage of Th2 cells as compared to old non-ovx females (t-test, p<0.05) (Fig 6H).

**Fig 7:**
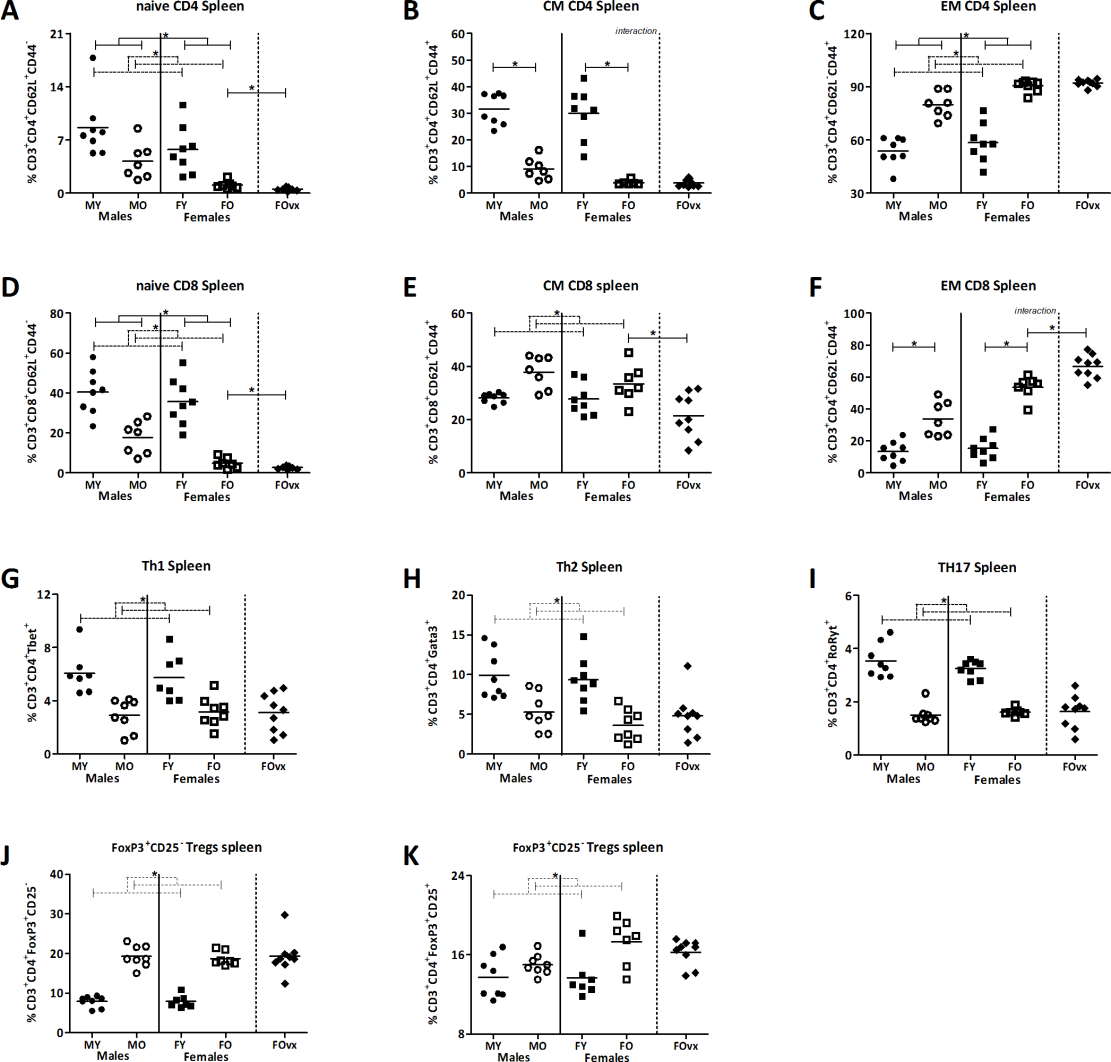
Effect of age and sex on T cell differentiation in the spleens. Percentage of CD62L^+^CD44^-^ naive CD4^+^ (A) and CD8^+^ (D), CD62L^+^CD44^+^ central memory CD4^+^ (B) and CD8^+^ (E), CD62L^-^CD44^+^ effector memory CD4^+^ (C) and CD8^+^ (F), T helper 1 (G), T helper 2 (H), T helper 17 (I), FoxP3^+^CD25^-^ T regulatory cells (J), and FoxP3^+^CD25^+^ T regulatory cells (K) in the spleen of young (3 months) and old (19 months) male and female B6 mice. T helper and T cytotoxic cells are expressed as the frequency of CD4^+^ and CD8^+^ cells within the CD3^+^ population respectively. Results are expressed as dot plots + means and were tested using Two-way ANOVA, followed by a Bonferroni post-test for comparison between groups. Significant age effects are indicated with dashed lines and significant sex effects are indicated with solid lines (p<0.05). An additional group of ovariectomized (ovx) old females was added and compared with the old females with a t-test to determine the effect of a loss of female sex hormones (human menopause).

In the spleen, old mice had a lower percentage of naïve CD4^+^ and CD8^+^ cells than young mice, while males had a higher percentage of naïve CD4^+^ and CD8^+^ cells than females (TWA, p<0.05) (Fig 7A and 7D). However, similar as in the PP, in the CM CD4^+^ population the effect of age was not the same in males and females (TWA, p<0.05). Ageing decreased the percentage of CM CD4^+^ cells in both males and females, however this effect was more pronounced in females (Bonferroni, p<0.05). Ageing increased the percentage of CM CD8^+^ cells, while sex had no effect on this population (TWA, p<0.05) (Fig 7B and 7E). Ageing also increased the percentage of EM CD4^+^ cells, while females had a higher percentage of EM CD4^+^ cells than males (TWA, p<0.05). The effect of age on the EM CD8^+^ population was different in males and females (TWA, p<0.05). Ageing increased the percentage of EM CD8^+^ cells in both males and females, but this effect was more pronounced in females (Bonferroni, p<0.05) (Fig 7C and 7F). Ovariectomy reduced the percentage of naïve CD4^+^ and CD8^+^ cells, CM CD8^+^ cells, while increased the percentage of EM CD8^+^ cells in the spleen (Fig 7A–7F).

Similar as within the PP, age had an effect on the percentage of T helper subsets in the spleen, while sex did not. Old mice had a lower percentage of Th1, Th2 and Th17 cells than young mice (Fig 7G–7I), while old mice had a higher percentage of FoxP3^+^CD25^-^ and FoxP3^+^CD25^+^ Tregs than young mice (TWA, p<0.05) (Fig 7J and 7K). Ovariectomy had no effect on the T helper subsets in the spleen.

We also looked at the percentage of B cells (CD19^+^B220^+^) in the PP and spleen, and the percentage of B cells expressing IgA (S6 Fig). Age and sex had no effect on the percentage of B cells in the PP. However, in the spleen males had a higher percentage of B cells than females. In both sexes, ageing increased the percentage of cells expressing IgA in the PP, while in the spleen this effect of age was only present in females.

### 3.5. Ageing and sex did not influence dendritic cell development in the Peyer’s patches

Dendritic cells (DCs), present just below the surface of the epithelial cells in the small intestine constantly sample the gut lumen. Upon encountering an antigen, DCs are activated and migrate to the PP or mesenteric lymph nodes to communicate with lymphocytes [32,33]. Depending on the type of antigen, they either activate or suppress an immune response by differentiating lymphocytes into effector cells.[33] Dendritic cells (DCs) in the PP expressing CD103 are able to differentiate T helper (Th) cells into FoxP3^+^ T regulatory cells (Tregs) [34].

**Fig 8:**
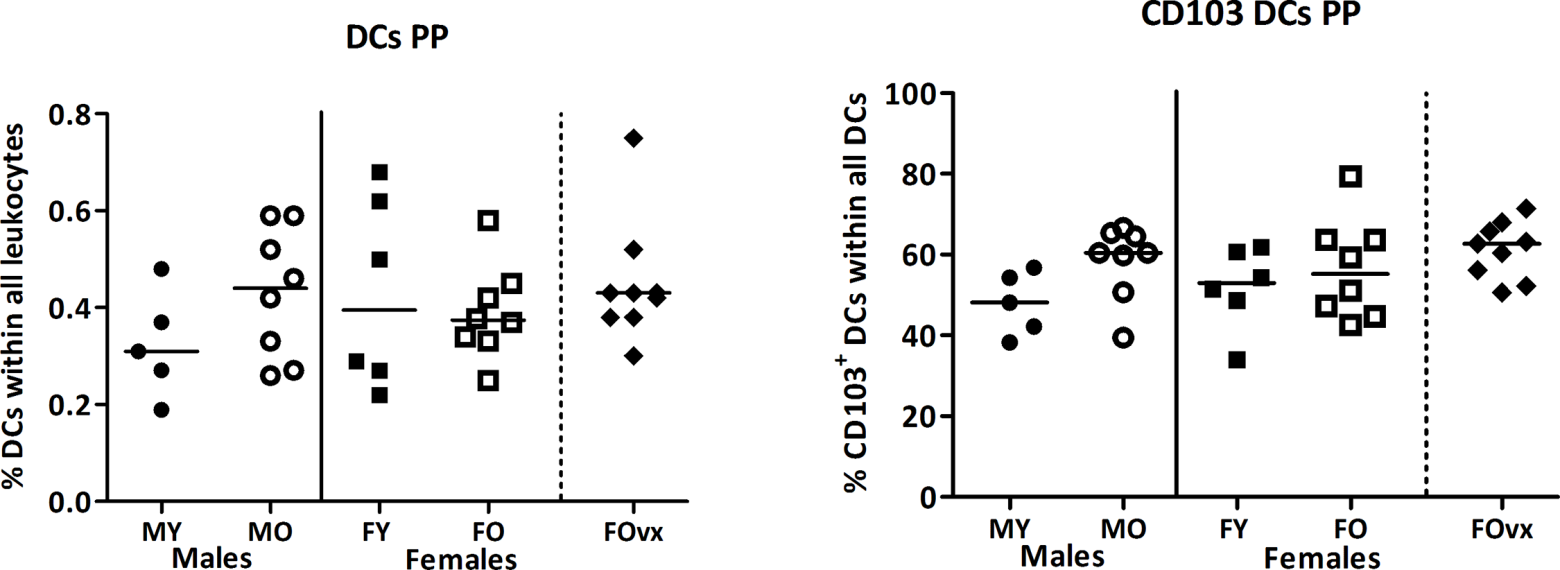
Effect of age and sex on the percentage of dendritic cells in the Peyer’s patches. Percentage of MHC2^+^CD64^-^CD19^-^CD11c^+^ dendritic cells (DCs) (A) and their expression of CD103 (B) in the Peyer’s patches (PP) of young (3 months) and old (19 months) male and female B6 mice. First leukocytes cells were selected based on size in the forward side scatter plot. DCs are expressed as the percentage MHC2^+^CD64^-^CD19^-^CD11c^+^ cells within all leukocytes. Results are expressed as dot plots + means and were tested using Two-way ANOVA followed by a Bonferroni post-test for comparison between groups. Significant age effects are indicated with dashed lines and significant sex effects are indicated with solid lines (p<0.05). An additional group of ovariectomized (ovx) old females was added and compared with the old females with a t-test to determine the effect of a loss of female sex hormones (human menopause).

Overall, age and sex had no effect on the percentage of DCs or the percentage of DCs expressing CD103 in the PP (TWA, p<0.05) (Fig 8). Ovariectomy also had no effect on the DC populations in the PP.

In the spleen, overall old mice had a lower percentage of DCs than young mice (TWA, p<0.05). The expression of CD103 on DCs in the spleen was lower than in the PP (approximately 10%), and the percentage of DCs expressing CD103 was overall higher in old mice than young mice (TWA, p<0.05). Sex had no overall effect on the percentage of DC, but females had an overall higher percentage of CD103^+^ DCs than males (TWA, p<0.05) (data not shown).

## 4. Discussion

In this study we showed that age influenced mucus properties, the intestinal microbiota composition and intestinal immunity in a sex dependent way. Both old male and old female mice had a decreased mucus thickness in the colon as compared to young mice and contact between the colon epithelium and microbiota was observed at several locations in the old mice, illustrating inadequacies in the barrier function of mucus layer in both sexes in old age. This deterioration of mucus may be due to a decreased mucus production such as decreased production of MUC1 or by an age dependent decrease in genes involved in mucus biosynthesis, such as *Chst2*, *Chst4* and *St3gal4*. This may result in mucus with different composition such as different sulfonation. Decreased mucus production has been associated with increased colonization with pathobionts [12,17,18], which we also observed in the old mice. We found, compared to young mice, a higher abundance of the potential pathobiont *Bacteroides vulgatus et rel*.in the old mice [35]. These changes in mucus and microbiota were associated with enhanced activation of the immune system as illustrated by higher numbers of CD4^+^ effector cells in old mice and lower numbers of naïve CD4^+^ and CD8^+^ T cells in the old mice.

Interactions between the microbiota and the intestinal barrier can have a significant impact on health [36,37]. For example, mice with an impaired mucus layer, as found in mice lacking the MUC2 mucin, develop colitis [17,38]. Here we showed that the colonic mucus layer becomes thinner and less effective as a physical barrier during ageing in both males and females. Old mice showed a significant shrinkage of the colonic mucus layer as compared to young mice, which was associated with bacterial penetration and direct contact of bacteria with the epithelium. The reduction in mucus thickness may therefore explain the gradual deterioration of barrier function in the elderly [19], but also the age induced changes in immune responses [39]. Indeed, our study also showed changes in intestinal immune responses with ageing. Such age induced changes in mucus and intestinal immunology may also be related to the finding of Ha *et al*., who found age-associated variables in the management of IBD in older IBD patients. Additional, a decline in mucus thickness may also play a role in the higher susceptibility for rheumatoid arthritis (RA) in RA susceptible *0401 mice, as these mice also showed an altered microbiota composition and had a significantly higher gut permeability as compared to naive mice [40].

Apart from the thickness of the mucus layer, we also investigated the expression of essential genes involved in goblet cell secretory processes and mucus biosynthesis in both ileum and colonic tissue. Several genes involved in mucus pathways were influenced by age and generally downregulated in old mice. We mainly found differences in genes involved in the later stage of mucus production, at which sulfation groups are added to the carbohydrate chains in the trans-Golgi apparatus by sulfotransferases (such as *Chst4*) [41–43], and where sialyl groups are added by sialyltransferases (such as *St3gal4-5*) [43]. Although no difference in the colonic mucus thickness between old male and females was found, most obvious changes induced by ageing in the colon were found in male mice. Of the mucins, *Muc1* was downregulated in old males as compared to young males, while in old females *Muc6* tended to be upregulated as compared to young females. The old mice may compensate for the loss of mucus thickness with the production of several anti-microbial peptides, such as *Reg4*, as this gene was upregulated. Although, old ovx females tended to have a thicker mucus layer than non-ovx females, ovariectomy seemed to have no effect on the mucus properties, as in old ovx females no genes related to mucus properties were differently expressed as compared to old non-ovx females.

The differences in mucus layer between young and old mice may be related to the observed differences in microbiota composition. Indeed, we found age related differences in the microbiota composition: aged mice had a higher abundance of the potential pathobiont *Bacteroides vulgatus et rel*. [35] than young mice. We also found a significantly lower abundance of various *Lactobacillus* species and unclassified *Clostridiales type IV* and *XIV* in old mice, of which the latter two are known butyrate producers. These species have beneficial (gastrointestinal) health effects [44,45], and a decrease in these species and an increase in potential pathobionts may be associated with the general higher susceptibility for diseases at an older age. Additionally, the change in microbiota composition may be related to the decreased mucus thickness in old mice, as for instance *Bacteriodetes vulgatus* has been shown to be able to degrade mucus [46]. However, from the present study, it cannot be concluded whether differences in microbiota induce differences in mucus thickness or the other way around. Further studies are needed to evaluate the underlying mechanisms. This could include studies measuring the mucus thickness at several time-point during the process of ageing, to investigate the development of mucus shrinkage. Additionally, studying mucus properties in the small intestine would be interesting as this is an important immunological site [47].

Differences in mucus thickness and composition as well as differences in the microbiota may induce differences in immune responses. Therefore we first performed a gene array on ileum and colonic tissue and indeed found that many immunological functions were affected by age, and sometimes dependent on sex. In both the ileum and colon, more functions related to immunity were influenced by age in males as compared to females. Many of these functions were related to lymphocyte numbers and function, such as activation of T lymphocytes, proliferation of T lymphocytes and differentiation of lymphocytes. Therefore, we also studied T lymphocyte subpopulations in the PP and in the spleen. We used the PP since it is an important immune sampling site of antigens from the gut lumen [31] and the spleen as a reference for the peripheral immune system. Both sexes showed typical signs of immune senescence [39], as a lower percentage of total T cells and naïve T cells and a higher percentage of effector memory T cells were found in old than in young mice in both organs [39,48]. This has been suggested to cause the lack of capacity of elderly to react to new pathogens. Furthermore, aged mice of both sexes had an increased percentage of Th17 cells in their PP, indicating an increased inflammation in old mice as compared with young mice. As Th17 cells are thought to play a central role in the induction and persistence of the inflammation in IBD [49], these cells may also be involved in the change in symptoms of IBD with ageing [6]. However, in the spleen, we found decreased percentages of Th17 cells. This is in contrast to other studies, showing an increased systemical Th17 response with ageing [50,51]. The reason for this is unknown, but it may be due to study object (mouse vs. human), mouse strain, age, and organ choice and marker selection (basal transcription factor expression in our study vs. cytokines induced after stimulation in other studies). In several immune populations, interaction between age and sex was present, especially in the maturation of T cells, suggesting that the ageing of immune cells is different between males and females. Interestingly, the effect of age was often more pronounced in females as compared to males, while the opposite was true for the immunological functions related to the gene expression in the ileum and colon.

## 5. Conclusions

The firm mucus layer in the colon was thinner in old mice than in young mice and was influenced by sex. The decline in mucus thickness during ageing was severe enough to allow contact between the epithelium and microbiota in both sexes. The decline in mucus thickness with ageing might be explained by downregulation of genes involved in mucus biosynthesis pathways and was associated with a changed microbiota, such as a relative high abundance of certain pathobionts and a relative low abundance of more beneficial bacteria strains. The age differences in mucus thickness, mucus gene expression and microbiota composition may have induced the age differences in immune responses in the PP, such as a decreased percentage of naïve T cells, increased percentage of effector memory cells and increased Th17 cells.

## Supporting information

S1 Fig Gating strategy for determination of T cell subsets in the Peyer’s patches. Lymphocytes were gated based on size in the forward side scatter plot (100.000 events are shown) and T cells were determined by selecting CD3^+^ cells. Within the CD3^+^ cells, CD8^+^ and CD4^+^ cells were detected. Within both the CD8^+^ and CD4^+^ population, the percentage of CD69, CD62L and CD44 positive cells was evaluated. CD62L^+^CD44^-^ are indicated as naïve cells, CD62L^+^CD44^+^ are indicated as central memory (CM) cells and CD62L^-^CD44^+^ are indicated as effector memory (EM) cells. Isotype controls are shown in panel B.
(TIF)

S2 Fig Gating strategy for the determination of T helper cells in the Peyer’s patches. Lymphocytes were gated on bases of their size in the forward side scatter plot (400.000 events are shown) and CD3^+^ T cells were selected. Next, T helper cells were selected by gating CD4^+^ cells. Within this CD4^+^ population, the percentage of cells expressing Tbet, Gata3, RORɣt, FoxP3 and CD25 was assessed. Isotype controls are shown in panel B.
(TIF)

S3 Fig Gating strategy for the determination of dendritic cells in the Peyer’s patches. First leukocytes were selected based on size in the forward side scatter plot (400.000 events are shown). DCs are selected as MHC2^+^CD64^-^CD19^-^CD11c^+^ cells. Within the DC population the expression of CD103 was determined. Isotype controls are shown in panel B.
(TIF)

S4 Fig The effect of age and sex on the MUC2 in the colon. Representative pictures of immunostaining of the MUC2 mucin (green) of young, old male, old female and old ovx female mice. Epithelial cells are indicated in blue. Scale bars: 50μm (A).
(TIF)

S5 Fig Effect of age on the abundance of several bacteria groups. Heat-maps showing the abundance of several bacteria that differed significantly between young (3 months) and old mice (19 months). A box with an asterisk (*) indicates bacteria which have a significantly different in that specific age group than in the other age group. Colors indicate relative abundances normalized per bacterial group (per row), dark blue is the lowest abundance and dark red the highest abundance detected over all the samples of a bacterial group.
(TIF)

S6 Fig Effect of age and sex on the percentage of B cells in the Peyer’s patches and spleen. Percentage of CD19^+^B220^+^ B cells in the Peyer’s patches (PP) (A) and in the spleen (B) and their expression of IgA (B&D) in young (3 months) and old (19 months) male and female B6 mice. First lymphocytes cells were selected based on size in the forward side scatter plot. B cells are expressed as the percentage CD19^+^B220^+^ cells within all lymphocytes. Results are expressed as dot plots + means and were tested using Two-way ANOVA followed by a Bonferroni post-test for comparison between groups. Significant age effects are indicated with dashed lines and significant sex effects are indicated with solid lines (p<0.05). An additional group of ovariectomized (ovx) old females was added and compared with the old females with a t-test to determine the effect of a loss of female sex hormones (human menopause).
(TIF)

S1 Table Weight of mice. (DOCX)

S2 Table Antibody specifications. (DOCX)

S3 Table Significant differences between old and young and between males and females (old males (MO), young males (MY), old females (FO) young females (FY) and ovariectomized females (FOvx)) in the expression of a selection of genes involved in the production of mucus, anti-microbial peptides (AMP) and tight junctions in the distal ileum. Only probe sets with a fold-change of at least 1.2 (up/down) and a q-value < 0.05 were considered to be significantly different. Significant results and trends are highlighted in bold.
(DOCX)

S4 Table Significant differences between old and young and between males and females (old males (MO), young males (MY), old females (FO) young females (FY) and ovariectomized females (FOvx)) in the expression of a selection of genes involved in the production of mucus, anti-microbial peptides (AMP) and tight junctions in the proximal colon. Only probe sets with a fold-change of at least 1.2 (up/down) and a q-value < 0.05 were considered to be significantly different. Significant results and trends are highlighted in bold.
(DOCX)

S5 Table Selection of immunological functions that are related to the genes with a different expression in young (3 months) and old (19 months) mice in both male and female B6 mice in the distal ileum. A p-value of <0.05 was used. The z-score gives an indication of the activation or inhibition of the functions in old versus young mice.
(DOCX)

S6 Table Selection of immunological functions that are related to the genes with a different expression in young (3 months) and old (19 months) mice in both male and female B6 mice in the proximal colon. A p-value of <0.05 was used. The z-score gives an indication of the activation or inhibition of the functions in old versus young mice.
(DOCX)
