# Supplementary figures and images for: The effect of age on the intestinal mucus thickness, microbiota composition and immunity in relation to sex in mice
Source: PLoS One. 2017 Sep 12;12(9):e0184274. doi: 10.1371/journal.pone.0184274 (PMC5595324; doi:10.1371/journal.pone.0184274)

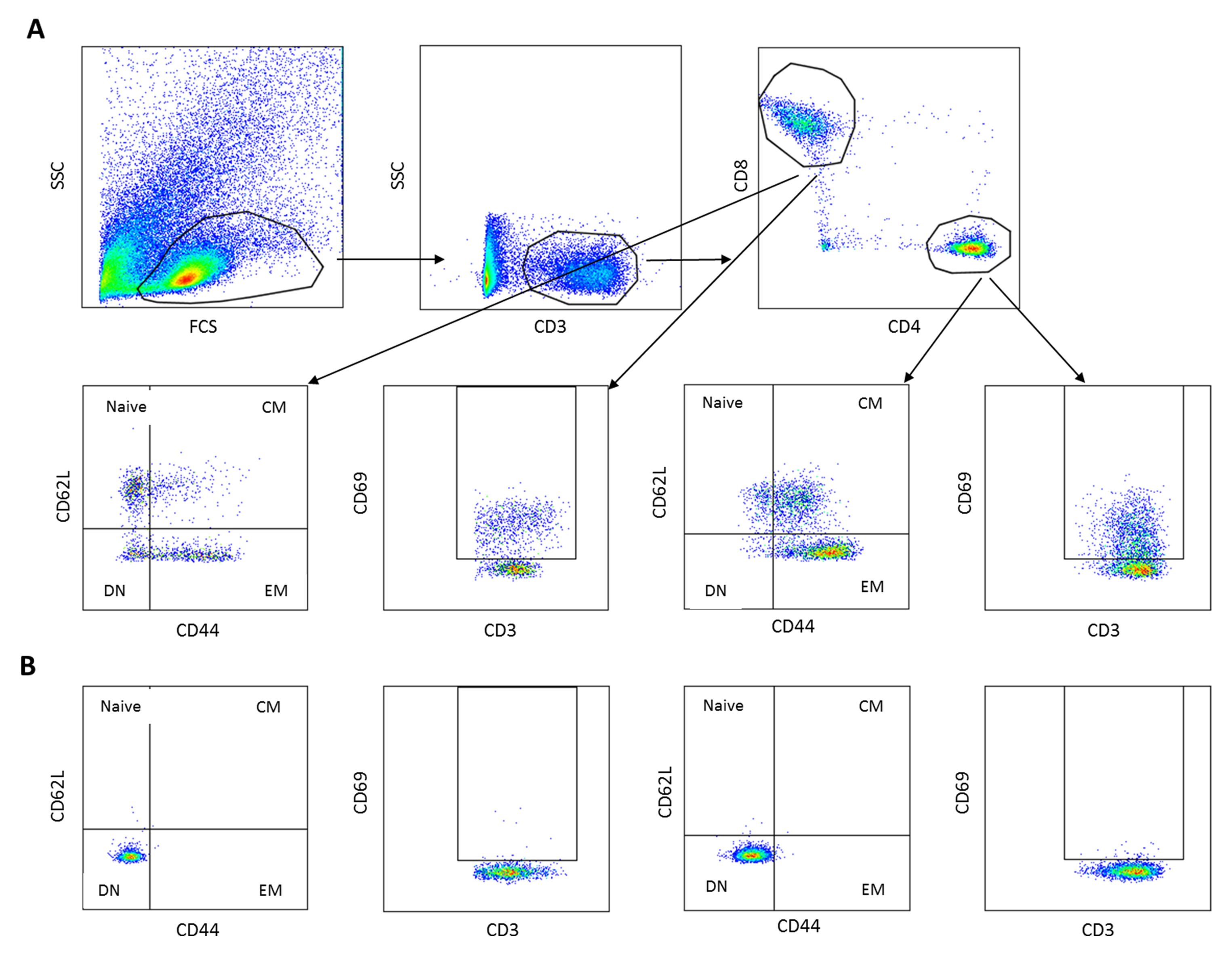

Supplement: S1 Fig — Lymphocytes were gated based on size in the forward side scatter plot (100.000 events are shown) and T cells were determined by selecting CD3+ cells. Within the CD3+ cells, CD8+ and CD4+ cells were detected. Within both the CD8+ and CD4+ population, the percentage of CD69, CD62L and CD44 positive cells was evaluated. CD62L+CD44- are indicated as naïve cells, CD62L+CD44+ are indicated as central memory (CM) cells and CD62L-CD44+ are indicated as effector memory (EM) cells. Isotype controls are shown in panel B. (TIF) [file pone.0184274.s001.tif]

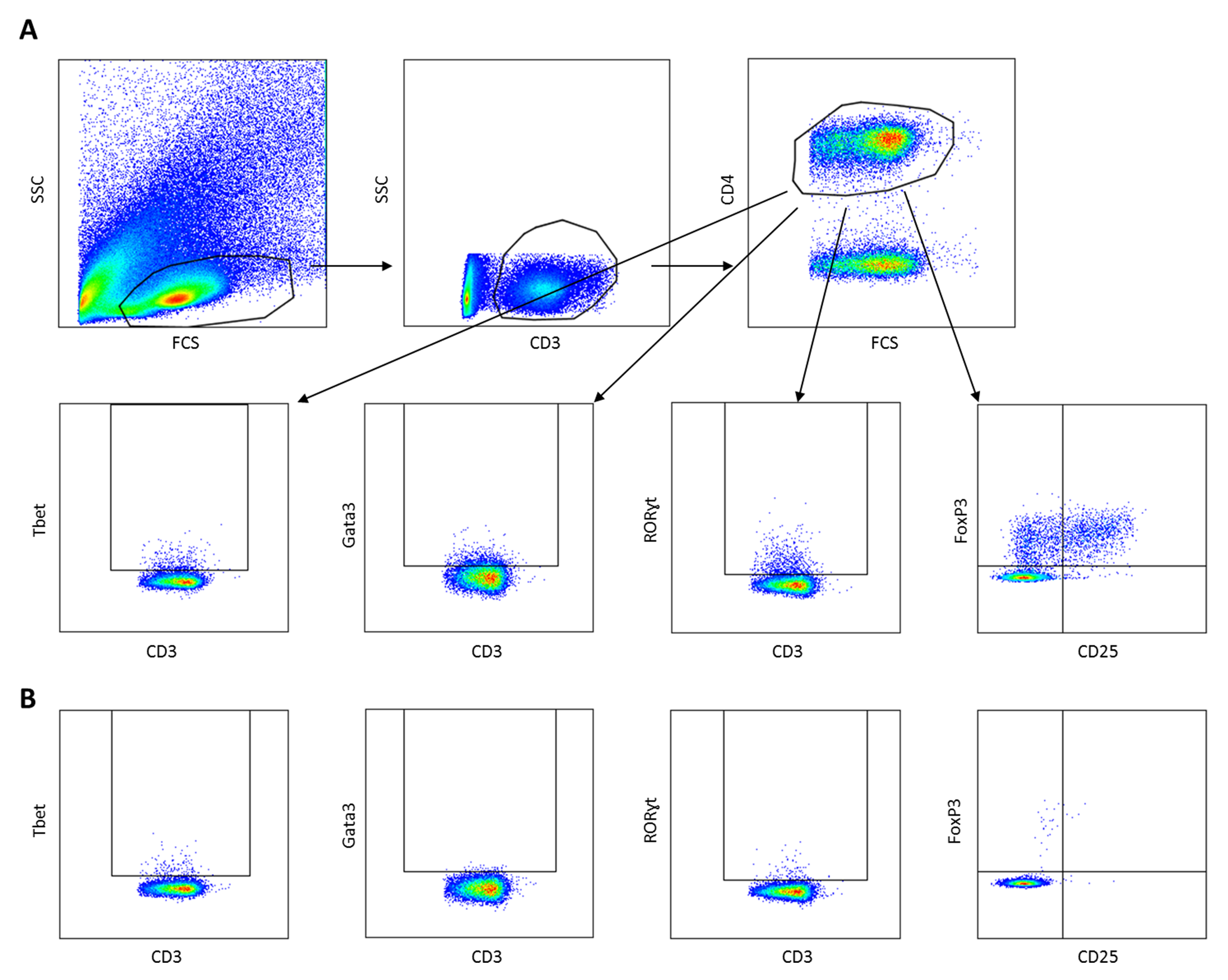

Supplement: S2 Fig — Lymphocytes were gated on bases of their size in the forward side scatter plot (400.000 events are shown) and CD3+ T cells were selected. Next, T helper cells were selected by gating CD4+ cells. Within this CD4+ population, the percentage of cells expressing Tbet, Gata3, RORɣt, FoxP3 and CD25 was assessed. Isotype controls are shown in panel B. (TIF) [file pone.0184274.s002.tif]

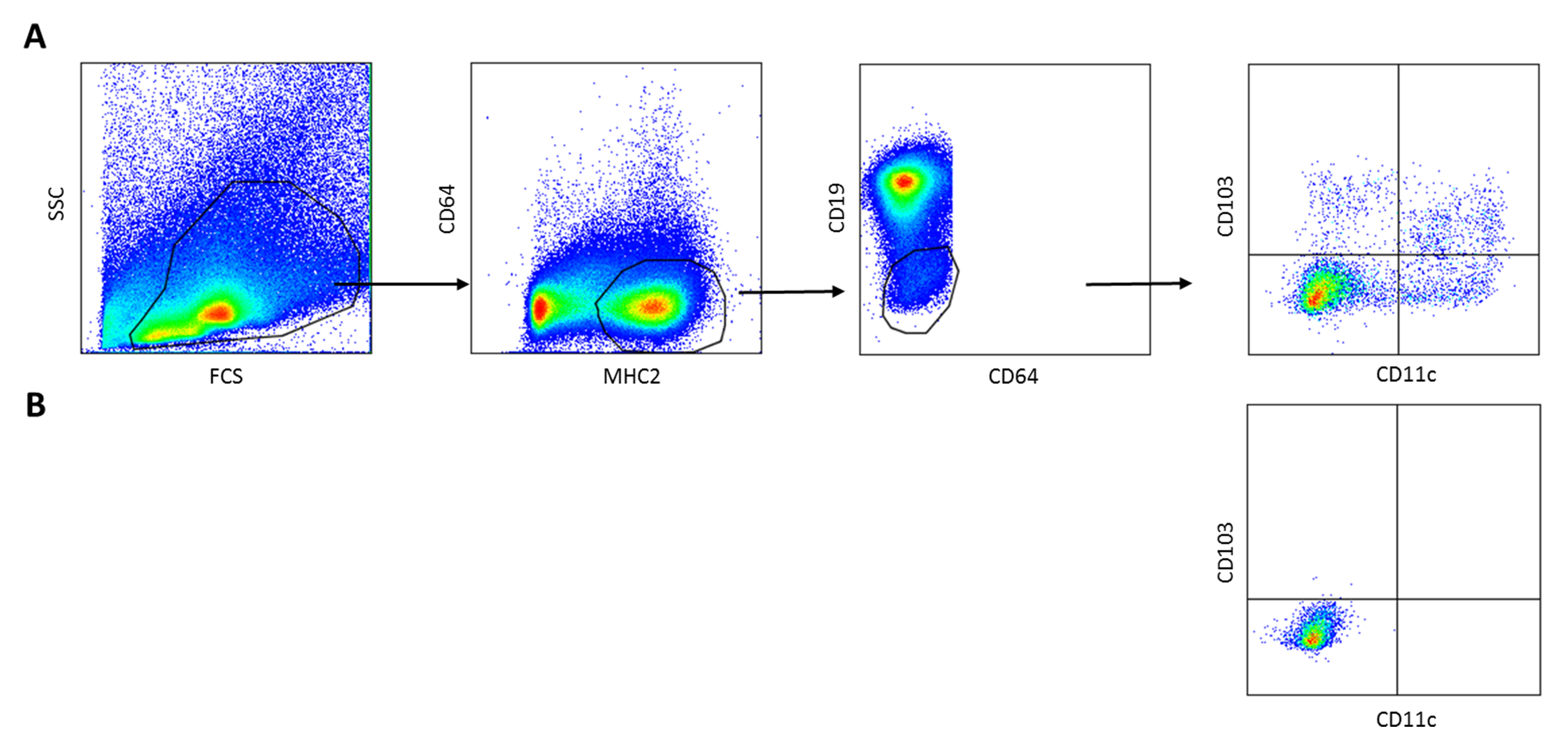

Supplement: S3 Fig — First leukocytes were selected based on size in the forward side scatter plot (400.000 events are shown). DCs are selected as MHC2+CD64-CD19-CD11c+ cells. Within the DC population the expression of CD103 was determined. Isotype controls are shown in panel B. (TIF) [file pone.0184274.s003.tif]

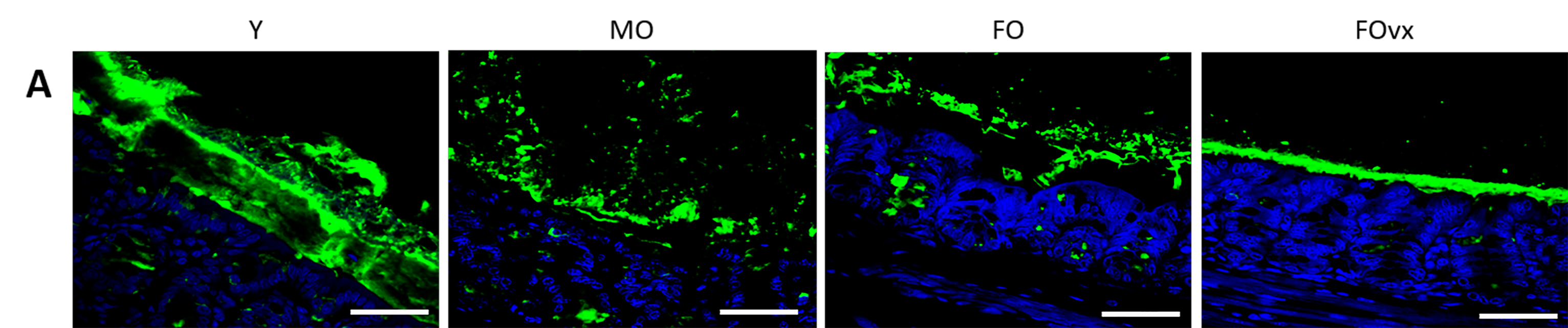

Supplement: S4 Fig — Representative pictures of immunostaining of the MUC2 mucin (green) of young, old male, old female and old ovx female mice. Epithelial cells are indicated in blue. Scale bars: 50μm (A). (TIF) [file pone.0184274.s004.tif]

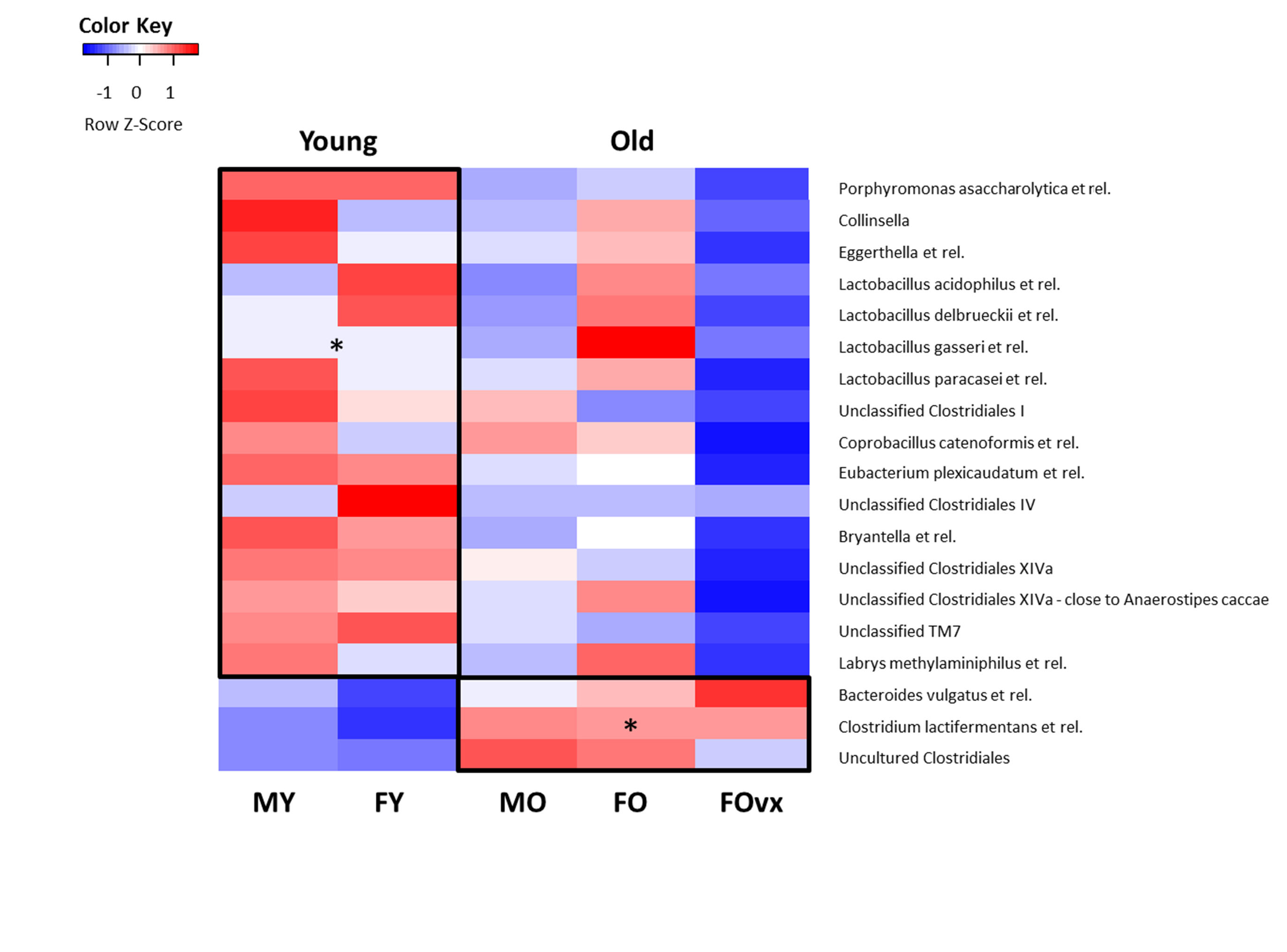

Supplement: S5 Fig — Heat-maps showing the abundance of several bacteria that differed significantly between young (3 months) and old mice (19 months). A box with an asterisk (*) indicates bacteria which have a significantly different in that specific age group than in the other age group. Colors indicate relative abundances normalized per bacterial group (per row), dark blue is the lowest abundance and dark red the highest abundance detected over all the samples of a bacterial group. (TIF) [file pone.0184274.s005.tif]

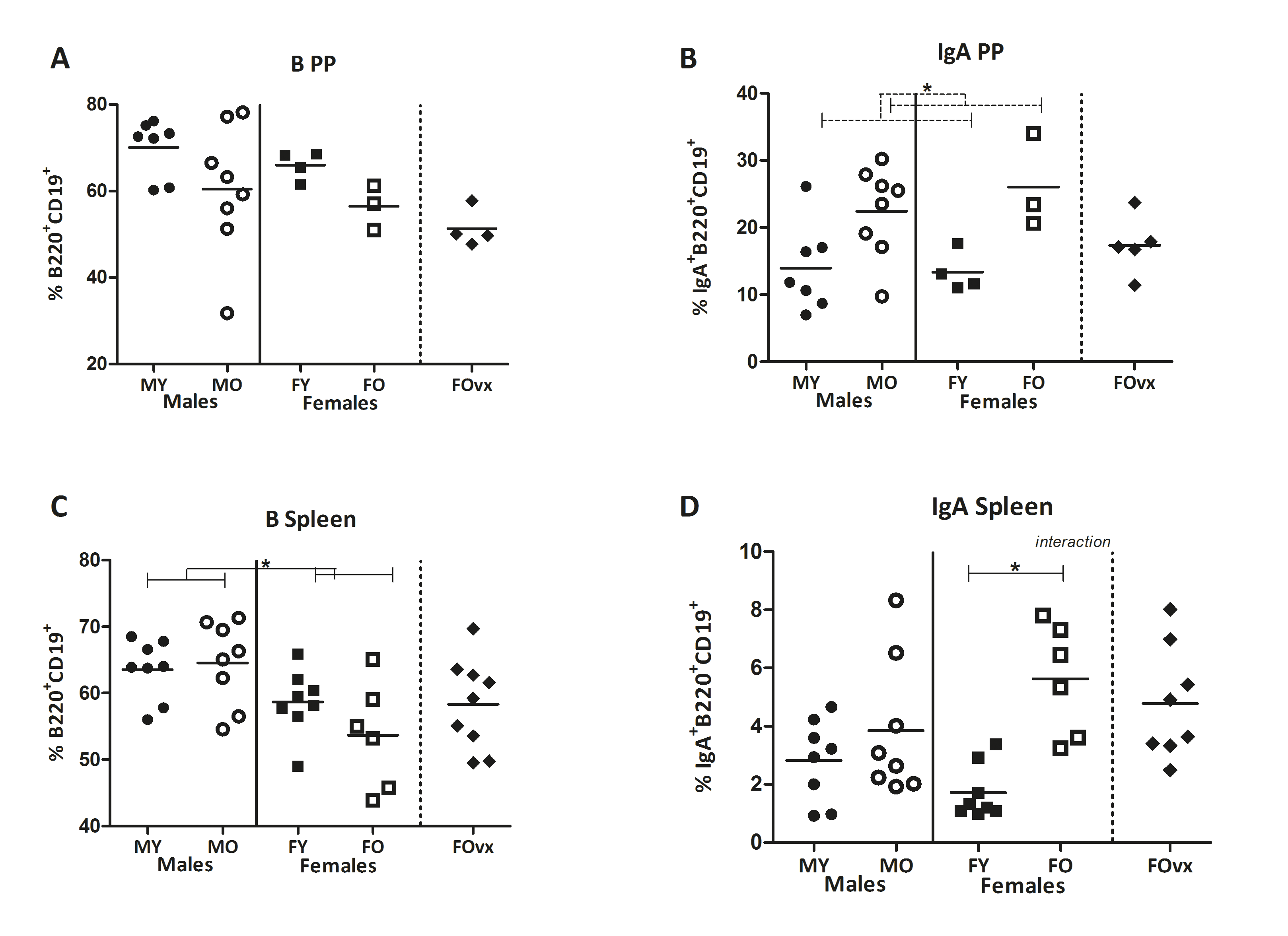

Supplement: S6 Fig — Percentage of CD19+B220+ B cells in the Peyer’s patches (PP) (A) and in the spleen (B) and their expression of IgA (B&D) in young (3 months) and old (19 months) male and female B6 mice. First lymphocytes cells were selected based on size in the forward side scatter plot. B cells are expressed as the percentage CD19+B220+ cells within all lymphocytes. Results are expressed as dot plots + means and were tested using Two-way ANOVA followed by a Bonferroni post-test for comparison between groups. Significant age effects are indicated with dashed lines and significant sex effects are indicated with solid lines (p<0.05). An additional group of ovariectomized (ovx) old females was added and compared with the old females with a t-test to determine the effect of a loss of female sex hormones (human menopause). (TIF) [file pone.0184274.s006.tif]
