# Supplementary material for: The effect of age on the intestinal mucus thickness, microbiota composition and immunity in relation to sex in mice
Source: PLoS One. 2017 Sep 12;12(9):e0184274. doi: 10.1371/journal.pone.0184274 (PMC5595324; doi:10.1371/journal.pone.0184274)
Supplement: S1 Table — (DOCX) [file pone.0184274.s007.docx]

S1 Table. Weight of mice

| **Weight in grams** | **Males** | **Females** | **Ovx females** |
| --- | --- | --- | --- |
| Young | 27.5^a^ | 19.7^b^ |  |
| Old | 41.5^c^ | 36.7^d^ | 39.5^d^ |

*The letters a-d indicate which groups differ significantly (t-test<0.05) from each other*
