# Supplementary material for: The effect of age on the intestinal mucus thickness, microbiota composition and immunity in relation to sex in mice
Source: PLoS One. 2017 Sep 12;12(9):e0184274. doi: 10.1371/journal.pone.0184274 (PMC5595324; doi:10.1371/journal.pone.0184274)
Supplement: S2 Table — (DOCX) [file pone.0184274.s008.docx]

**S2 Table.** Antibody specifications.

| **Specificity** | **Host species** | **Clone name** | **Fluorchrome** | **Concentration** | **Dilution*^a^*** | **Supplier** |
| --- | --- | --- | --- | --- | --- | --- |
| CD3 | Rat | 17A2 | FitC | 0.5 mg/ml | 50x | Biolegend |
| CD4 | Rat | GK1.5 | PerCP-Cy5.5 | 0.2 mg/ml | 75x | Biolegend |
| Tbet | Mouse | 4B10 | BV421 | 0.08 mg/ml | 10x | Biolegend |
| RORɣt | Rat | B2D | PE | 0.2 mg/ml | 100x | eBioscience |
| Gata3 | Mouse | L50-823 | AF647 | 0.2 mg/ml | 100x | BDbiosciences |
| CD3 | Rat | 17A2 | BV421 | 0.2 mg/ml | 50x | Biolegend |
| FoxP3 | Rat | FJK-16s | FitC | 0.5 mg/ml | 50x | eBioscience |
| CD25 | Rat | PC61 | PE-Cy7 | 0.2 mg/ml | 50x | Biolegend |
| CD8 | Rat | 53-6.7 | PerCP-Cy5.5 | 0.2 mg/ml | 50x | Biolegend |
| CD4 | Rat | GK1.5 | PE-Cy7 | 0.2 mg/ml | 100x | Biolegend |
| CD69 | Armenian Hamster | H1.2F3 | FitC | 0.5 mg/ml | 25x | Biolegend |
| CD62L | Rat | MEL-14 | BV605 | 0.2 mg/ml | 25x | Biolegend |
| CD44 | Rat | IM7 | APC-Cy7 | 0.2 mg/ml | 100x | Biolegend |
| CD19 | Rat | 6D5 | BV605 | 0.2 mg/ml | 25x | Biolegend |
| B220 | Rat | RA-6B2 | BV421 | 0.2 mg/ml | 15x | Biolegend |
| IgA | Rat | C10-3 | FitC | 0.5 mg/ml | 50x | BDbiosciences |
| MHC2 | Rat | M5/114.15.2 | PerCP-Cy5.5 | 0.2 mg/ml | 200x | Biolegend |
| CD64 | Mouse | X54-5/7.1 | PE | 0.2 mg/ml | 25x | Biolegend |
| CD19 | Rat | 6D5 | BV605 | 0.2 mg/ml | 25x | Biolegend |
| CD11c | Armenian Hamster | N418 | PE-Cy7 | 0.2 mg/ml | 100x | Biolegend |
| CD103 | Armenian Hamster | 2E7 | BV421 | 0.2 mg/ml | 20x | Biolegend |

*^a^* *Dilution used in a total volume of 25 µl supplemented with PBS + 10% FCS*
