# Supplementary material for: The effect of age on the intestinal mucus thickness, microbiota composition and immunity in relation to sex in mice
Source: PLoS One. 2017 Sep 12;12(9):e0184274. doi: 10.1371/journal.pone.0184274 (PMC5595324; doi:10.1371/journal.pone.0184274)
Supplement: S3 Table — Only probe sets with a fold-change of at least 1.2 (up/down) and a q-value < 0.05 were considered to be significantly different. Significant results and trends are highlighted in bold. (DOCX) [file pone.0184274.s009.docx]

**S3 Table.** Significant differences between old and young and between males and females (old males (MO), young males (MY), old females (FO) young females (FY) and ovariectomized females (FOvx)) in the expression of a selection of genes involved in the production of mucus, anti-microbial peptides (AMP) and tight junctions in the distal ileum. Only probe sets with a fold-change of at least 1.2 (up/down) and a q-value < 0.05 were considered to be significantly different. Significant results and trends are highlighted in bold.

| **gene name** | **Fold Change**  **MO vs MY** | **q-value**  **MO vs MY** | **Fold Change**  **FO vs FY** | **q-value**  **FO vs FY** | **Fold Change**  **FO vs FOvx** | **q-value**  **FO vs FOvx** |
| --- | --- | --- | --- | --- | --- | --- |
| **Mucus** |  |  |  |  |  |  |
| Muc1 | -1,15 | 0,510 | -1,00 | 0,640 | -1,00 | 1,000 |
| Muc13 | -1,05 | 0,663 | 1,08 | 0,198 | 1,02 | 1,000 |
| Muc15 | 1,13 | 0,369 | 1,09 | 0,255 | 1,11 | 1,000 |
| Muc19 | 1,00 | 0,852 | -1,04 | 0,414 | 1,03 | 1,000 |
| Muc2 | 1,10 | 0,182 | 1,03 | 0,466 | -1,01 | 1,000 |
| Muc20 | 1,05 | 0,707 | -1,02 | 0,572 | 1,13 | 1,000 |
| Muc3 | 1,01 | 0,795 | -1,00 | 0,637 | -1,02 | 1,000 |
| Muc4 | 1,01 | 0,848 | 1,34 | 0,120 | 1,11 | 1,000 |
| Muc5ac | 1,09 | 0,757 | 1,32 | 0,162 | 1,31 | 1,000 |
| Muc5b | 1,01 | 0,843 | 1,06 | 0,366 | 1,05 | 1,000 |
| Muc6 | 1,10 | 0,790 | 1,40 | 0,232 | 1,41 | 1,000 |
| Mucl1 | -1,05 | 0,722 | -1,06 | 0,425 | -1,02 | 1,000 |
| **Mucus biosynthesis** |  |  |  |  |  |  |
| Chst1 | 1,02 | 0,835 | 1,00 | 0,644 | -1,07 | 1,000 |
| Chst10 | 1,02 | 0,825 | 1,10 | 0,310 | 1,01 | 1,000 |
| Chst11 | -1,05 | 0,750 | 1,06 | 0,457 | 1,04 | 1,000 |
| Chst12 | -1,09 | 0,689 | 1,16 | 0,217 | 1,03 | 1,000 |
| Chst13 | 1,00 | 0,850 | 1,02 | 0,589 | 1,16 | 1,000 |
| Chst14 | -1,01 | 0,841 | -1,04 | 0,539 | -1,06 | 1,000 |
| Chst15 | -1,19 | 0,056 | 1,05 | 0,351 | -1,02 | 1,000 |
| Chst2 | -1,15 | 0,432 | 1,11 | 0,283 | -1,07 | 1,000 |
| Chst3 | -1,41 | 0,577 | 1,05 | 0,603 | -1,06 | 1,000 |
| Chst4 | 1,08 | 0,720 | -1,04 | 0,555 | 1,14 | 1,000 |
| Chst5 | 1,08 | 0,650 | -1,05 | 0,467 | -1,04 | 1,000 |
| Chst7 | 1,02 | 0,821 | -1,12 | 0,261 | -1,13 | 1,000 |
| Chst8 | 1,07 | 0,733 | **-1,33** | **0,047** | -1,19 | 1,000 |
| Chst9 | -1,04 | 0,776 | 1,04 | 0,529 | 1,02 | 1,000 |
| Gal3st1 | 1,09 | 0,655 | -1,02 | 0,593 | -1,05 | 1,000 |
| Gal3st3 | 1,00 | 0,854 | -1,02 | 0,581 | 1,03 | 1,000 |
| Gal3st4 | -1,28 | 0,166 | 1,04 | 0,540 | 1,06 | 1,000 |
| St3gal4 | -1,14 | 0,104 | -1,04 | 0,358 | 1,03 | 1,000 |
| St3gal5 | -1,15 | 0,709 | 1,11 | 0,494 | 1,17 | 1,000 |
| St6galnac1 | -1,03 | 0,822 | -1,12 | 0,371 | -1,06 | 1,000 |
| St6galnac2 | -1,04 | 0,769 | -1,08 | 0,343 | -1,02 | 1,000 |
| St6galnac3 | 1,02 | 0,835 | 1,04 | 0,543 | -1,12 | 1,000 |
| St6galnac4 | 1,16 | 0,388 | 1,05 | 0,505 | -1,06 | 1,000 |
| St6galnac5 | 1,09 | 0,635 | 1,08 | 0,383 | 1,07 | 1,000 |
| St6galnac6 | 1,53 | 0,432 | 1,51 | 0,186 | -1,03 | 1,000 |
| Klf4 | 1,03 | 0,779 | -1,09 | 0,268 | -1,05 | 1,000 |
| Retnlb | -1,66 | 0,729 | 2,13 | 0,366 | 1,45 | 1,000 |
| Tff1 | 1,07 | 0,811 | 1,41 | 0,184 | 1,38 | 1,000 |
| Tff2 | -1,08 | 0,805 | 1,21 | 0,407 | 1,40 | 1,000 |
| Tff3 | 1,15 | 0,324 | **-1,21** | **0,033** | -1,08 | 1,000 |
| Fut1 | -1,04 | 0,795 | 1,12 | 0,338 | -1,00 | 1,000 |
| Fut10 | -1,04 | 0,756 | 1,07 | 0,351 | -1,01 | 1,000 |
| Fut11 | 1,02 | 0,785 | -1,02 | 0,575 | -1,06 | 1,000 |
| Fut2 | -1,39 | 0,735 | 1,60 | 0,396 | 1,28 | 1,000 |
| Fut4 | 1,08 | 0,622 | -1,15 | 0,117 | -1,05 | 1,000 |
| Fut7 | 1,11 | 0,652 | 1,08 | 0,456 | 1,12 | 1,000 |
| Fut8 | 1,06 | 0,663 | 1,09 | 0,229 | 1,05 | 1,000 |
| Fut9 | 1,03 | 0,778 | -1,03 | 0,533 | 1,11 | 1,000 |
| Itgb1 | -1,09 | 0,391 | 1,10 | 0,095 | 1,03 | 1,000 |
| Itgb1bp1 | -1,06 | 0,648 | 1,10 | 0,217 | -1,05 | 1,000 |
| Itgb1bp2 | -1,07 | 0,688 | 1,10 | 0,333 | 1,04 | 1,000 |
| Itgb2 | -1,13 | 0,678 | 1,08 | 0,508 | -1,06 | 1,000 |
| Itgb2l | 1,07 | 0,652 | 1,02 | 0,596 | -1,00 | 1,000 |
| Itgb3 | -1,06 | 0,712 | 1,16 | 0,156 | 1,03 | 1,000 |
| Itgb3bp | -1,04 | 0,797 | -1,06 | 0,494 | 1,08 | 1,000 |
| Itgb4 | 1,03 | 0,722 | -1,11 | 0,073 | 1,02 | 1,000 |
| Itgb5 | -1,02 | 0,784 | 1,11 | 0,144 | 1,03 | 1,000 |
| Itgb6 | -1,11 | 0,679 | -1,03 | 0,596 | 1,11 | 1,000 |
| Itgb7 | -1,16 | 0,637 | -1,09 | 0,485 | -1,05 | 1,000 |
| Itgb8 | -1,01 | 0,842 | 1,03 | 0,579 | -1,10 | 1,000 |
| Itgbl1 | -1,04 | 0,779 | 1,03 | 0,540 | 1,07 | 1,000 |
| **Tight junction genes** |  |  |  |  |  |  |
| Ocln | 1,10 | 0,543 | -1,03 | 0,523 | -1,01 | 1,000 |
| Cldn1 | 1,05 | 0,810 | 1,26 | 0,193 | -1,15 | 1,000 |
| Cldn10 | 1,05 | 0,743 | 1,14 | 0,220 | -1,06 | 1,000 |
| Cldn11 | -1,06 | 0,705 | 1,01 | 0,621 | -1,02 | 1,000 |
| Cldn12 | 1,07 | 0,601 | -1,02 | 0,577 | 1,03 | 1,000 |
| Cldn14 | 1,07 | 0,664 | 1,13 | 0,180 | 1,03 | 1,000 |
| Cldn15 | 1,09 | 0,465 | 1,05 | 0,382 | -1,04 | 1,000 |
| Cldn16 | 1,07 | 0,697 | -1,00 | 0,642 | -1,01 | 1,000 |
| Cldn17 | 1,04 | 0,757 | -1,11 | 0,206 | 1,07 | 1,000 |
| Cldn18 | 1,01 | 0,846 | 1,08 | 0,303 | 1,08 | 1,000 |
| Cldn19 | 1,05 | 0,735 | 1,07 | 0,407 | 1,02 | 1,000 |
| Cldn2 | 1,01 | 0,848 | **-1,31** | **0,014** | -1,09 | 1,000 |
| Cldn3 | -1,01 | 0,835 | **-1,23** | **0,011** | -1,07 | 1,000 |
| Cldn4 | 1,22 | 0,428 | -1,24 | 0,144 | -1,03 | 1,000 |
| Cldn5 | -1,09 | 0,623 | 1,02 | 0,580 | -1,06 | 1,000 |
| Cldn6 | 1,05 | 0,755 | 1,14 | 0,241 | 1,05 | 1,000 |
| Cldn7 | -1,04 | 0,634 | -1,19 | 0,002 | 1,01 | 1,000 |
| Cldn8 | 1,14 | 0,717 | -1,05 | 0,590 | -1,01 | 1,000 |
| Cldn9 | -1,11 | 0,623 | -1,04 | 0,553 | 1,09 | 1,000 |
| Esam | -1,01 | 0,825 | 1,13 | 0,087 | -1,01 | 1,000 |
| Icam1 | -1,11 | 0,623 | -1,06 | 0,495 | -1,07 | 1,000 |
| Icam2 | -1,10 | 0,663 | 1,05 | 0,516 | 1,03 | 1,000 |
| Pecam1 | -1,09 | 0,587 | 1,15 | 0,125 | -1,03 | 1,000 |
| F11r | -1,07 | 0,452 | -1,16 | 0,009 | -1,02 | 1,000 |
| Igsf5 | 1,02 | 0,778 | 1,01 | 0,616 | 1,05 | 1,000 |
| Jam2 | 1,15 | 0,336 | **1,29** | **0,009** | -1,05 | 1,000 |
| Jam3 | 1,03 | 0,789 | **1,26** | **0,032** | 1,00 | 1,000 |
| Actn1 | 1,06 | 0,659 | 1,13 | 0,093 | -1,04 | 1,000 |
| Actn2 | -1,11 | 0,648 | 1,05 | 0,509 | 1,02 | 1,000 |
| Actn3 | 1,12 | 0,497 | 1,15 | 0,132 | 1,05 | 1,000 |
| Actn4 | -1,04 | 0,634 | -1,01 | 0,556 | -1,01 | 1,000 |
| Ctnna1 | -1,01 | 0,834 | -1,08 | 0,050 | -1,02 | 1,000 |
| Ctnna2 | -1,02 | 0,827 | **1,21** | **0,048** | -1,14 | 1,000 |
| Ctnna3 | -1,04 | 0,798 | **1,33** | **0,058** | 1,00 | 1,000 |
| Ctnnb1 | -1,02 | 0,738 | 1,00 | 0,613 | -1,00 | 1,000 |
| Cgn | -1,09 | 0,487 | -1,08 | 0,242 | 1,04 | 1,000 |
| Cttn | -1,06 | 0,458 | -1,11 | 0,023 | -1,01 | 1,000 |
| Epb4.1 | -1,01 | 0,845 | 1,02 | 0,541 | 1,03 | 1,000 |
| Hcls1 | -1,13 | 0,674 | 1,03 | 0,598 | -1,08 | 1,000 |
| Inadl | 1,04 | 0,703 | 1,02 | 0,578 | 1,00 | 1,000 |
| Magi1 | 1,00 | 0,854 | 1,02 | 0,561 | -1,02 | 1,000 |
| Magi3 | 1,02 | 0,793 | 1,02 | 0,507 | 1,03 | 1,000 |
| Mllt4 | 1,02 | 0,713 | 1,03 | 0,411 | 1,02 | 1,000 |
| Mpdz | 1,06 | 0,728 | **1,43** | **0,005** | 1,02 | 1,000 |
| Pard3 | -1,00 | 0,844 | 1,01 | 0,562 | 1,02 | 1,000 |
| Sympk | 1,01 | 0,815 | -1,07 | 0,200 | -1,04 | 1,000 |
| Tjap1 | -1,06 | 0,634 | -1,13 | 0,096 | -1,10 | 1,000 |
| Tjp1 | 1,03 | 0,720 | -1,04 | 0,350 | -1,02 | 1,000 |
| Tjp2 | 1,02 | 0,708 | 1,02 | 0,475 | 1,01 | 1,000 |
| Tjp3 | 1,01 | 0,816 | -1,12 | 0,094 | -1,06 | 1,000 |
| Vapa | -1,01 | 0,822 | -1,01 | 0,553 | 1,07 | 1,000 |
| **Anti-microbial peptides** |  |  |  |  |  |  |
| Def6 | -1,13 | 0,585 | -1,05 | 0,522 | -1,01 | 1,000 |
| Def8 | 1,24 | 0,240 | -1,10 | 0,359 | -1,05 | 1,000 |
| Defa24 | 1,01 | 0,835 | -1,04 | 0,489 | 1,02 | 1,000 |
| Defb1 | -1,26 | 0,751 | 1,49 | 0,359 | 1,14 | 1,000 |
| Defb10 | -1,12 | 0,433 | -1,04 | 0,519 | -1,13 | 1,000 |
| Defb11 | 1,01 | 0,838 | -1,04 | 0,535 | 1,06 | 1,000 |
| Defb12 | 1,03 | 0,836 | -1,11 | 0,509 | -1,20 | 1,000 |
| Defb13 | -1,02 | 0,818 | 1,04 | 0,484 | 1,05 | 1,000 |
| Defb14 | 1,04 | 0,735 | -1,08 | 0,263 | 1,01 | 1,000 |
| Defb15 | 1,03 | 0,803 | -1,07 | 0,456 | 1,03 | 1,000 |
| Defb18 | 1,07 | 0,597 | 1,00 | 0,647 | 1,04 | 1,000 |
| Defb19 | 1,03 | 0,767 | 1,03 | 0,500 | 1,04 | 1,000 |
| Defb2 | -1,09 | 0,643 | 1,03 | 0,570 | -1,00 | 1,000 |
| Defb20 | 1,04 | 0,722 | -1,01 | 0,599 | 1,04 | 1,000 |
| Defb21 | -1,03 | 0,793 | 1,15 | 0,143 | 1,18 | 1,000 |
| Defb22 | 1,07 | 0,562 | -1,13 | 0,079 | 1,03 | 1,000 |
| Defb23 | -1,01 | 0,840 | 1,00 | 0,645 | -1,01 | 1,000 |
| Defb25 | 1,03 | 0,803 | 1,06 | 0,461 | 1,05 | 1,000 |
| Defb26 | 1,02 | 0,824 | -1,11 | 0,305 | 1,02 | 1,000 |
| Defb28 | -1,06 | 0,712 | -1,13 | 0,190 | -1,04 | 1,000 |
| Defb29 | -1,01 | 0,828 | -1,07 | 0,384 | 1,01 | 1,000 |
| Defb3 | -1,03 | 0,811 | -1,15 | 0,178 | -1,04 | 1,000 |
| Defb30 | 1,01 | 0,848 | 1,00 | 0,643 | 1,09 | 1,000 |
| Defb33 | 1,03 | 0,805 | 1,02 | 0,602 | 1,04 | 1,000 |
| Defb34 | -1,02 | 0,832 | 1,07 | 0,475 | 1,14 | 1,000 |
| Defb35 | -1,07 | 0,777 | 1,10 | 0,466 | -1,05 | 1,000 |
| Defb36 | 1,13 | 0,458 | -1,04 | 0,514 | 1,01 | 1,000 |
| Defb37 | 1,40 | 0,648 | -1,09 | 0,585 | -1,18 | 1,000 |
| Defb38 | 1,02 | 0,813 | -1,09 | 0,248 | -1,08 | 1,000 |
| Defb39 | 1,36 | 0,616 | -1,13 | 0,540 | -1,23 | 1,000 |
| Defb4 | -1,01 | 0,851 | -1,02 | 0,608 | -1,05 | 1,000 |
| Defb40 | 1,24 | 0,701 | 1,19 | 0,472 | -1,02 | 1,000 |
| Defb41 | -1,13 | 0,534 | -1,13 | 0,250 | 1,03 | 1,000 |
| Defb42 | 1,05 | 0,718 | 1,06 | 0,390 | 1,09 | 1,000 |
| Defb43 | -1,07 | 0,661 | -1,09 | 0,288 | -1,07 | 1,000 |
| Defb44-ps | 1,03 | 0,784 | -1,11 | 0,189 | 1,02 | 1,000 |
| Defb45 | -1,05 | 0,689 | -1,06 | 0,349 | -1,01 | 1,000 |
| Defb46 | 1,05 | 0,749 | -1,07 | 0,457 | 1,10 | 1,000 |
| Defb47 | 1,04 | 0,761 | 1,15 | 0,144 | 1,17 | 1,000 |
| Defb48 | -1,04 | 0,733 | -1,00 | 0,636 | -1,04 | 1,000 |
| Defb5 | 1,17 | 0,569 | 1,04 | 0,571 | 1,23 | 1,000 |
| Defb50 | 1,02 | 0,808 | 1,04 | 0,504 | 1,06 | 1,000 |
| Defb6 | -1,20 | 0,339 | 1,01 | 0,611 | 1,02 | 1,000 |
| Defb7 | 1,01 | 0,848 | -1,04 | 0,532 | 1,02 | 1,000 |
| Defb8 | 1,06 | 0,743 | 1,01 | 0,618 | 1,09 | 1,000 |
| Defb9 | -1,03 | 0,769 | -1,05 | 0,368 | 1,04 | 1,000 |
| Defb9 | -1,03 | 0,769 | -1,05 | 0,368 | 1,04 | 1,000 |
| Reg1 | -1,02 | 0,831 | **-1,30** | **0,072** | -1,19 | 1,000 |
| Reg2 | -1,03 | 0,801 | 1,03 | 0,538 | -1,04 | 1,000 |
| Reg3a | 1,07 | 0,791 | -1,20 | 0,321 | -1,09 | 1,000 |
| Reg3b | -1,08 | 0,640 | -1,03 | 0,555 | -1,01 | 1,000 |
| Reg3d | -1,05 | 0,785 | -1,22 | 0,152 | -1,10 | 1,000 |
| Reg3g | -1,18 | 0,510 | -1,10 | 0,410 | -1,08 | 1,000 |
| Reg4 | 1,72 | 0,171 | 1,26 | 0,328 | -1,03 | 1,000 |
