# Supplementary material for: The effect of age on the intestinal mucus thickness, microbiota composition and immunity in relation to sex in mice
Source: PLoS One. 2017 Sep 12;12(9):e0184274. doi: 10.1371/journal.pone.0184274 (PMC5595324; doi:10.1371/journal.pone.0184274)
Supplement: S4 Table — Only probe sets with a fold-change of at least 1.2 (up/down) and a q-value < 0.05 were considered to be significantly different. Significant results and trends are highlighted in bold. (DOCX) [file pone.0184274.s010.docx]

**S4 Table.** Significant differences between old and young and between males and females (old males (MO), young males (MY), old females (FO) young females (FY) and ovariectomized females (FOvx)) in the expression of a selection of genes involved in the production of mucus, anti-microbial peptides (AMP) and tight junctions in the proximal colon. Only probe sets with a fold-change of at least 1.2 (up/down) and a q-value < 0.05 were considered to be significantly different. Significant results and trends are highlighted in bold.

| **gene name** | **Fold Change**  **MO vs MY** | **q-value**  **MO vs MY** | **Fold Change**  **FO vs FY** | **q-value**  **FO vs FY** | **Fold Change**  **FO vs FOvx** | | **q-value**  **FO vs FOvx** | |
| --- | --- | --- | --- | --- | --- | --- | --- | --- |
| **Mucus** |  |  |  |  |  |  | |  |
| *Muc1* | **-1,59** | **0,021** | -1,21 | 0,264 | 1,15 | 0,665 | |  |
| *Muc13* | -1,03 | 0,456 | 1,03 | 0,386 | 1,01 | 0,795 | |  |
| *Muc15* | -1,06 | 0,486 | 1,10 | 0,259 | -1,01 | 0,826 | |  |
| *Muc19* | 1,01 | 0,651 | -1,02 | 0,522 | -1,05 | 0,704 | |  |
| *Muc2* | -1,04 | 0,341 | -1,01 | 0,541 | 1,04 | 0,624 | |  |
| *Muc20* | -1,02 | 0,662 | 1,08 | 0,404 | -1,03 | 0,797 | |  |
| *Muc3* | 1,02 | 0,568 | -1,01 | 0,574 | -1,01 | 0,833 | |  |
| *Muc4* | -1,03 | 0,671 | 1,25 | 0,211 | 1,25 | 0,523 | |  |
| *Muc5ac* | 1,00 | 0,699 | 1,05 | 0,388 | 1,01 | 0,839 | |  |
| *Muc5b* | -1,06 | 0,452 | 1,04 | 0,444 | 1,02 | 0,806 | |  |
| *Muc6* | -1,06 | 0,592 | **1,35** | **0,073** | 1,07 | 0,770 | |  |
| *Mucl1* | 1,17 | 0,122 | 1,03 | 0,539 | 1,10 | 0,591 | |  |
| **Mucus biosynthesis** |  |  |  |  |  |  | |  |
| *Chst1* | 1,17 | 0,138 | 1,04 | 0,506 | -1,03 | 0,800 | |  |
| *Chst10* | 1,01 | 0,680 | 1,03 | 0,550 | -1,07 | 0,679 | |  |
| *Chst11* | 1,05 | 0,607 | -1,04 | 0,557 | -1,06 | 0,775 | |  |
| *Chst12* | -1,11 | 0,328 | -1,18 | 0,130 | -1,13 | 0,559 | |  |
| *Chst13* | 1,11 | 0,302 | 1,16 | 0,143 | 1,09 | 0,618 | |  |
| *Chst14* | -1,08 | 0,484 | -1,12 | 0,304 | -1,08 | 0,709 | |  |
| *Chst15* | -1,15 | 0,147 | -1,18 | 0,076 | -1,03 | 0,789 | |  |
| *Chst2* | **-1,22** | **0,094** | -1,08 | 0,384 | 1,09 | 0,663 | |  |
| *Chst3* | -1,15 | 0,271 | -1,04 | 0,519 | -1,01 | 0,833 | |  |
| *Chst4* | **-1,46** | **0,010** | -1,08 | 0,435 | 1,20 | 0,474 | |  |
| *Chst5* | -1,07 | 0,485 | 1,00 | 0,624 | -1,06 | 0,733 | |  |
| *Chst7* | -1,07 | 0,445 | -1,02 | 0,577 | -1,09 | 0,641 | |  |
| *Chst8* | 1,07 | 0,495 | -1,08 | 0,380 | -1,04 | 0,784 | |  |
| *Chst9* | 1,06 | 0,462 | 1,06 | 0,385 | -1,02 | 0,796 | |  |
| *Gal3st1* | 1,10 | 0,368 | -1,05 | 0,471 | 1,09 | 0,665 | |  |
| *Gal3st3* | -1,17 | 0,094 | 1,01 | 0,594 | -1,01 | 0,829 | |  |
| *Gal3st4* | -1,03 | 0,655 | 1,09 | 0,426 | 1,07 | 0,756 | |  |
| *St3gal4* | **-2,00** | **0,005** | -1,37 | 0,148 | -1,08 | 0,784 | |  |
| *St3gal5* | **2,15** | **0,077** | 1,89 | 0,116 | -1,27 | 0,715 | |  |
| *St6galnac1* | 1,25 | 0,158 | -1,10 | 0,412 | -1,13 | 0,635 | |  |
| *St6galnac2* | -1,09 | 0,211 | -1,08 | 0,223 | 1,10 | 0,464 | |  |
| *St6galnac3* | 1,00 | 0,707 | -1,05 | 0,432 | 1,01 | 0,836 | |  |
| *St6galnac4* | 1,02 | 0,658 | 1,00 | 0,623 | 1,31 | 0,317 | |  |
| *St6galnac5* | -1,07 | 0,568 | -1,17 | 0,267 | 1,02 | 0,829 | |  |
| *St6galnac6* | -1,17 | 0,208 | 1,01 | 0,619 | 1,30 | 0,317 | |  |
| *Klf4* | 1,13 | 0,203 | 1,09 | 0,275 | 1,01 | 0,842 | |  |
| *Retnlb* | -1,39 | 0,481 | 1,38 | 0,409 | -1,15 | 0,801 | |  |
| *Tff1* | 1,06 | 0,472 | 1,06 | 0,379 | -1,11 | 0,535 | |  |
| *Tff2* | -1,03 | 0,633 | 1,13 | 0,257 | 1,01 | 0,839 | |  |
| *Tff3* | 1,09 | 0,233 | -1,05 | 0,381 | -1,03 | 0,772 | |  |
| *Fut1* | -1,00 | 0,707 | -1,02 | 0,595 | -1,07 | 0,767 | |  |
| *Fut10* | -1,14 | 0,135 | -1,06 | 0,395 | -1,05 | 0,711 | |  |
| *Fut11* | 1,04 | 0,475 | -1,10 | 0,125 | -1,03 | 0,755 | |  |
| *Fut2* | -1,29 | 0,120 | -1,02 | 0,585 | 1,26 | 0,447 | |  |
| *Fut4* | **-1,37** | **0,079** | **-1,38** | **0,060** | 1,10 | 0,714 | |  |
| *Fut7* | -1,07 | 0,529 | -1,03 | 0,556 | -1,20 | 0,464 | |  |
| *Fut8* | -1,17 | 0,081 | -1,11 | 0,192 | 1,05 | 0,720 | |  |
| *Fut9* | -1,02 | 0,673 | 1,06 | 0,493 | 1,11 | 0,665 | |  |
| *Itgb1* | -1,10 | 0,012 | -1,02 | 0,433 | -1,02 | 0,710 | |  |
| *Itgb1bp1* | 1,07 | 0,392 | -1,06 | 0,384 | -1,03 | 0,788 | |  |
| *Itgb1bp2* | -1,02 | 0,652 | -1,06 | 0,462 | -1,11 | 0,615 | |  |
| *Itgb2* | 1,15 | 0,194 | 1,09 | 0,325 | -1,14 | 0,508 | |  |
| *Itgb2l* | -1,04 | 0,563 | 1,02 | 0,577 | 1,13 | 0,517 | |  |
| *Itgb3* | -1,01 | 0,677 | -1,13 | 0,232 | 1,03 | 0,802 | |  |
| *Itgb3bp* | -1,10 | 0,487 | -1,14 | 0,336 | -1,01 | 0,840 | |  |
| *Itgb4* | -1,03 | 0,542 | -1,04 | 0,409 | 1,07 | 0,574 | |  |
| *Itgb5* | 1,04 | 0,534 | 1,01 | 0,597 | -1,03 | 0,757 | |  |
| *Itgb6* | 1,05 | 0,571 | 1,17 | 0,187 | 1,03 | 0,808 | |  |
| *Itgb7* | 1,08 | 0,408 | 1,06 | 0,422 | -1,23 | 0,317 | |  |
| *Itgb8* | -1,18 | 0,170 | **-1,23** | **0,072** | -1,12 | 0,593 | |  |
| *Itgbl1* | 1,16 | 0,157 | 1,10 | 0,267 | -1,12 | 0,527 | |  |
| ***Tight junction genes*** |  |  |  |  |  |  | |  |
| *Ocln* | -1,07 | 0,318 | -1,04 | 0,444 | -1,07 | 0,609 | |  |
| *Cldn1* | 1,23 | 0,187 | -1,04 | 0,552 | -1,13 | 0,639 | |  |
| *Cldn10* | **1,21** | **0,051** | 1,04 | 0,502 | -1,04 | 0,749 | |  |
| *Cldn11* | -1,04 | 0,553 | **1,20** | **0,043** | 1,07 | 0,674 | |  |
| *Cldn12* | -1,14 | 0,067 | -1,03 | 0,488 | 1,01 | 0,827 | |  |
| *Cldn14* | 1,05 | 0,483 | 1,02 | 0,549 | -1,06 | 0,643 | |  |
| *Cldn15* | -1,07 | 0,210 | -1,07 | 0,190 | 1,03 | 0,716 | |  |
| *Cldn16* | -1,02 | 0,644 | 1,18 | 0,110 | 1,01 | 0,840 | |  |
| *Cldn17* | 1,12 | 0,331 | 1,15 | 0,195 | 1,04 | 0,791 | |  |
| *Cldn18* | 1,10 | 0,333 | 1,07 | 0,376 | 1,09 | 0,609 | |  |
| *Cldn19* | 1,01 | 0,674 | 1,03 | 0,486 | 1,11 | 0,459 | |  |
| *Cldn2* | -1,10 | 0,530 | -1,06 | 0,530 | 1,32 | 0,434 | |  |
| *Cldn3* | -1,04 | 0,452 | -1,11 | 0,058 | 1,02 | 0,786 | |  |
| *Cldn4* | 1,15 | 0,532 | **1,74** | **0,049** | 1,26 | 0,631 | |  |
| *Cldn5* | -1,22 | 0,187 | -1,14 | 0,313 | -1,34 | 0,342 | |  |
| *Cldn6* | -1,03 | 0,611 | 1,02 | 0,572 | -1,18 | 0,451 | |  |
| *Cldn7* | -1,05 | 0,406 | 1,02 | 0,493 | 1,01 | 0,823 | |  |
| *Cldn8* | -1,34 | 0,140 | -1,24 | 0,225 | 1,26 | 0,514 | |  |
| *Cldn9* | 1,10 | 0,302 | 1,06 | 0,386 | -1,01 | 0,840 | |  |
| *Esam* | 1,03 | 0,618 | 1,04 | 0,474 | -1,05 | 0,731 | |  |
| *Icam1* | -1,05 | 0,548 | 1,06 | 0,399 | 1,15 | 0,464 | |  |
| *Icam2* | -1,02 | 0,640 | -1,01 | 0,594 | -1,14 | 0,456 | |  |
| *Pecam1* | -1,04 | 0,500 | -1,07 | 0,278 | -1,10 | 0,489 | |  |
| *F11r* | -1,05 | 0,263 | -1,06 | 0,158 | -1,01 | 0,813 | |  |
| *Igsf5* | -1,02 | 0,575 | -1,03 | 0,431 | 1,01 | 0,810 | |  |
| *Jam2* | 1,16 | 0,081 | -1,09 | 0,222 | -1,12 | 0,468 | |  |
| *Jam3* | -1,16 | 0,233 | -1,20 | 0,132 | -1,07 | 0,730 | |  |
| *Actn1* | -1,08 | 0,246 | -1,13 | 0,049 | 1,00 | 0,849 | |  |
| *Actn2* | -1,34 | 0,244 | -1,18 | 0,389 | -1,03 | 0,836 | |  |
| *Actn3* | 1,13 | 0,271 | 1,05 | 0,486 | -1,09 | 0,650 | |  |
| *Actn4* | 1,06 | 0,237 | 1,10 | 0,065 | 1,06 | 0,539 | |  |
| *Ctnna1* | -1,01 | 0,557 | -1,04 | 0,161 | 1,00 | 0,851 | |  |
| *Ctnna2* | -1,07 | 0,472 | -1,19 | 0,103 | 1,00 | 0,845 | |  |
| *Ctnna3* | -1,06 | 0,479 | -1,14 | 0,154 | -1,01 | 0,836 | |  |
| *Ctnnb1* | -1,08 | 0,056 | -1,06 | 0,112 | 1,03 | 0,620 | |  |
| *Cgn* | -1,07 | 0,400 | 1,02 | 0,561 | 1,15 | 0,410 | |  |
| *Cttn* | 1,01 | 0,678 | 1,03 | 0,454 | 1,09 | 0,442 | |  |
| *Epb4.1* | 1,02 | 0,608 | -1,05 | 0,290 | 1,03 | 0,704 | |  |
| *Hcls1* | -1,04 | 0,594 | 1,02 | 0,583 | -1,12 | 0,592 | |  |
| *Inadl* | -1,03 | 0,548 | -1,11 | 0,092 | -1,01 | 0,818 | |  |
| *Magi1* | -1,18 | 0,006 | -1,16 | 0,013 | -1,02 | 0,763 | |  |
| *Magi3* | 1,02 | 0,575 | -1,00 | 0,611 | -1,02 | 0,788 | |  |
| *Mllt4* | -1,02 | 0,532 | -1,06 | 0,135 | 1,02 | 0,716 | |  |
| *Mpdz* | 1,04 | 0,545 | -1,05 | 0,450 | -1,08 | 0,609 | |  |
| *Pard3* | -1,03 | 0,459 | -1,04 | 0,318 | 1,03 | 0,689 | |  |
| *Sympk* | 1,02 | 0,618 | 1,02 | 0,469 | 1,11 | 0,355 | |  |
| *Tjap1* | -1,08 | 0,316 | -1,09 | 0,229 | 1,08 | 0,589 | |  |
| *Tjp1* | -1,03 | 0,532 | 1,01 | 0,605 | -1,03 | 0,742 | |  |
| *Tjp2* | -1,01 | 0,678 | 1,06 | 0,312 | 1,03 | 0,745 | |  |
| *Tjp3* | 1,08 | 0,151 | 1,01 | 0,568 | 1,07 | 0,457 | |  |
| *Vapa* | 1,00 | 0,703 | 1,05 | 0,227 | -1,02 | 0,741 | |  |
| ***Anti-microbial peptides*** |  |  |  |  |  |  | |  |
| *Def6* | 1,10 | 0,303 | -1,11 | 0,231 | -1,08 | 0,633 | |  |
| *Def8* | -1,01 | 0,694 | -1,06 | 0,397 | 1,03 | 0,794 | |  |
| *Defa24* | **4,55** | **0,044** | 1,29 | 0,511 | -2,52 | 0,492 | |  |
| *Defb1* | -1,01 | 0,659 | 1,01 | 0,613 | -1,06 | 0,639 | |  |
| *Defb10* | 1,01 | 0,678 | 1,04 | 0,398 | 1,04 | 0,719 | |  |
| *Defb11* | 1,12 | 0,254 | -1,01 | 0,611 | -1,00 | 0,846 | |  |
| *Defb12* | 1,00 | 0,707 | 1,11 | 0,515 | 1,40 | 0,553 | |  |
| *Defb13* | 1,04 | 0,593 | 1,03 | 0,547 | 1,04 | 0,773 | |  |
| *Defb14* | 1,08 | 0,378 | 1,03 | 0,519 | -1,02 | 0,809 | |  |
| *Defb15* | -1,07 | 0,541 | 1,03 | 0,578 | -1,09 | 0,713 | |  |
| *Defb18* | 1,11 | 0,249 | 1,01 | 0,613 | -1,03 | 0,782 | |  |
| *Defb19* | 1,03 | 0,613 | 1,12 | 0,157 | -1,05 | 0,705 | |  |
| *Defb2* | 1,04 | 0,590 | -1,10 | 0,312 | -1,18 | 0,448 | |  |
| *Defb20* | 1,03 | 0,597 | 1,13 | 0,157 | -1,01 | 0,839 | |  |
| *Defb21* | -1,03 | 0,622 | 1,11 | 0,247 | 1,02 | 0,813 | |  |
| *Defb22* | 1,08 | 0,329 | 1,01 | 0,593 | -1,10 | 0,527 | |  |
| *Defb23* | 1,03 | 0,643 | 1,02 | 0,596 | -1,03 | 0,817 | |  |
| *Defb25* | -1,04 | 0,607 | **1,21** | **0,099** | 1,04 | 0,792 | |  |
| *Defb26* | 1,11 | 0,303 | 1,01 | 0,612 | -1,07 | 0,693 | |  |
| *Defb28* | 1,02 | 0,641 | 1,11 | 0,276 | 1,00 | 0,848 | |  |
| *Defb29* | -1,00 | 0,697 | 1,05 | 0,466 | -1,09 | 0,603 | |  |
| *Defb3* | -1,04 | 0,611 | 1,21 | 0,101 | 1,05 | 0,772 | |  |
| *Defb30* | 1,08 | 0,458 | 1,01 | 0,601 | -1,15 | 0,492 | |  |
| *Defb33* | 1,00 | 0,703 | 1,06 | 0,459 | -1,08 | 0,703 | |  |
| *Defb34* | 1,11 | 0,376 | **1,29** | **0,040** | 1,07 | 0,729 | |  |
| *Defb35* | 1,16 | 0,256 | -1,02 | 0,592 | -1,17 | 0,509 | |  |
| *Defb36* | -1,02 | 0,629 | -1,09 | 0,295 | -1,06 | 0,711 | |  |
| *Defb37* | 1,30 | 0,244 | 1,19 | 0,343 | -1,01 | 0,842 | |  |
| *Defb38* | 1,01 | 0,677 | -1,09 | 0,174 | -1,04 | 0,686 | |  |
| *Defb39* | 1,07 | 0,375 | 1,14 | 0,088 | 1,04 | 0,738 | |  |
| *Defb4* | -1,11 | 0,343 | 1,19 | 0,108 | 1,13 | 0,525 | |  |
| *Defb40* | 1,36 | 0,151 | 1,30 | 0,186 | 1,09 | 0,759 | |  |
| *Defb41* | 1,02 | 0,641 | -1,02 | 0,558 | -1,09 | 0,624 | |  |
| *Defb42* | 1,05 | 0,590 | 1,07 | 0,450 | 1,04 | 0,799 | |  |
| *Defb43* | 1,19 | 0,056 | 1,04 | 0,470 | 1,00 | 0,845 | |  |
| *Defb44-ps* | 1,14 | 0,176 | 1,06 | 0,411 | 1,13 | 0,497 | |  |
| *Defb45* | -1,06 | 0,421 | 1,03 | 0,474 | -1,06 | 0,642 | |  |
| *Defb46* | 1,18 | 0,164 | 1,12 | 0,272 | 1,01 | 0,840 | |  |
| *Defb47* | -1,03 | 0,590 | -1,07 | 0,339 | -1,07 | 0,643 | |  |
| *Defb48* | 1,06 | 0,463 | 1,16 | 0,076 | 1,07 | 0,631 | |  |
| *Defb5* | 1,18 | 0,269 | 1,02 | 0,588 | 1,02 | 0,831 | |  |
| *Defb50* | **1,22** | **0,013** | 1,04 | 0,466 | 1,07 | 0,597 | |  |
| *Defb6* | 1,11 | 0,292 | 1,05 | 0,446 | -1,03 | 0,801 | |  |
| *Defb7* | 1,06 | 0,494 | 1,04 | 0,489 | -1,06 | 0,717 | |  |
| *Defb8* | 1,11 | 0,390 | 1,03 | 0,552 | 1,05 | 0,769 | |  |
| *Defb9* | 1,04 | 0,518 | 1,12 | 0,114 | 1,01 | 0,821 | |  |
| *Defb9* | 1,04 | 0,518 | 1,12 | 0,114 | 1,01 | 0,821 | |  |
| *Reg1* | -1,19 | 0,198 | -1,07 | 0,462 | 1,03 | 0,813 | |  |
| *Reg2* | 1,08 | 0,322 | 1,08 | 0,292 | 1,04 | 0,749 | |  |
| *Reg3a* | -1,17 | 0,480 | -1,11 | 0,494 | -1,03 | 0,836 | |  |
| *Reg3b* | -2,82 | 0,307 | -1,51 | 0,490 | -1,13 | 0,831 | |  |
| *Reg3d* | 1,09 | 0,282 | 1,03 | 0,523 | -1,01 | 0,834 | |  |
| *Reg3g* | -1,94 | 0,450 | 1,04 | 0,622 | -1,09 | 0,837 | |  |
| *Reg4* | **3,46** | **0,071** | **4,94** | **0,020** | -1,29 | 0,770 | |  |
