# Supplementary material for: The effect of age on the intestinal mucus thickness, microbiota composition and immunity in relation to sex in mice
Source: PLoS One. 2017 Sep 12;12(9):e0184274. doi: 10.1371/journal.pone.0184274 (PMC5595324; doi:10.1371/journal.pone.0184274)
Supplement: S5 Table — A p-value of <0.05 was used. The z-score gives an indication of the activation or inhibition of the functions in old versus young mice. (DOCX) [file pone.0184274.s011.docx]

**S5 Table.** Selection of immunological functions that are related to the genes with a different expression in young (3 months) and old (19 months) mice in both male and female B6 mice in the distal ileum. A p-value of <0.05 was used. The z-score gives an indication of the activation or inhibition of the functions in old versus young mice.

| **Diseases or Functions Annotation** | **z-score**  **MO vs MY** | **z-score**  **FO vs FY** | **p-Value**  **MO vs MY** | **p-Value**  **FO vs FY** |
| --- | --- | --- | --- | --- |
| activation of antigen presenting cells | -1.11 |  | 1.93E-05 |  |
| activation of leukocytes | -0.63 | 1.64 | 1.77E-16 | 9.10E-08 |
| activation of lymphocytes | -0.94 | 0.59 | 3.53E-14 | 1.81E-06 |
| activation of natural killer cells | -0.24 |  | 1.27E-08 |  |
| activation of phagocytes | -1.01 |  | 5.39E-07 |  |
| activation of T lymphocytes | -1.67 | -0.13 | 3.74E-14 | 1.52E-06 |
| adhesion of granulocytes | -0.32 |  | 4.72E-05 |  |
| adhesion of immune cells | -1.13 | 1.74 | 1.23E-07 | 4.40E-06 |
| cell proliferation of T lymphocytes | -0.16 | 0.72 | 1.12E-20 | 1.89E-07 |
| cell-mediated response | -1.95 |  | 1.70E-06 |  |
| development of helper T lymphocytes | -2.70 |  | 5.64E-05 |  |
| differentiation of B lymphocytes | -1.59 |  | 2.03E-05 |  |
| differentiation of helper T lymphocytes | -0.57 |  | 1.57E-06 |  |
| differentiation of T lymphocytes | -1.49 | 0.59 | 6.27E-14 | 1.09E-05 |
| function of cytotoxic T cells |  |  | 3.17E-05 |  |
| function of leukocytes | -2.18 | -1.05 | 1.77E-18 | 5.08E-07 |
| function of lymphocytes | -1.76 | -0.19 | 1.08E-11 | 6.26E-08 |
| function of phagocytes |  |  | 1.16E-07 |  |
| function of T lymphocytes | -1.52 | -0.38 | 1.05E-09 | 5.83E-06 |
| homing of leukocytes | -1.84 | 0.68 | 4.28E-09 | 3.36E-07 |
| homing of lymphocytes | -2.89 |  | 1.09E-05 |  |
| homing of T lymphocytes | -2.46 |  | 7.08E-06 |  |
| inflammatory response | 0.03 | 2.79 | 3.66E-10 | 3.99E-12 |
| interaction of antigen presenting cells | -2.08 |  | 3.23E-05 |  |
| interaction of lymphocytes | -2.42 | -0.07 | 4.96E-08 | 3.07E-06 |
| interaction of T lymphocytes | -3.07 | -0.67 | 2.49E-06 | 3.23E-06 |
| leukocyte migration | -2.94 | 0.83 | 5.13E-18 | 1.81E-13 |
| Lymphocyte homeostasis | -3.07 |  | 1.05E-17 |  |
| Lymphocyte migration | -3.81 | 0.15 | 7.83E-10 | 1.52E-06 |
| maturation of leukocytes | -2.78 |  | 6.37E-08 |  |
| maturation of lymphocytes | -2.09 |  | 1.26E-06 |  |
| maturation of T lymphocytes | -1.86 |  | 2.99E-08 |  |
| migration of phagocytes | -2.65 |  | 7.69E-06 |  |
| production of antibody | 1.16 | 0.04 | 8.92E-09 | 3.16E-06 |
| proliferation of B lymphocytes | -1.35 |  | 1.67E-06 |  |
| proliferation of immune cells | -1.17 | 0.74 | 5.20E-22 | 3.73E-09 |
| proliferation of lymphocytes | -1.05 | 0.63 | 6.03E-22 | 2.36E-09 |
| quantity of antigen presenting cells | 0.57 |  | 1.70E-06 |  |
| quantity of B lymphocytes | -2.43 |  | 1.33E-06 |  |
| quantity of CD4+ T-lymphocytes | -2.55 |  | 1.77E-11 |  |
| quantity of CD8+ T lymphocyte | -2.52 | 0.25 | 2.00E-13 | 6.04E-09 |
| quantity of dendritic cells | -0.07 |  | 4.82E-05 |  |
| quantity of granulocytes | 0.50 |  | 4.47E-06 |  |
| quantity of intraepithelial T lymphocytes | -1.35 |  | 1.85E-05 |  |
| quantity of leukocytes | -2.25 | -0.17 | 1.49E-26 | 3.62E-12 |
| quantity of lymphocytes | -3.32 | -0.25 | 1.12E-20 | 8.44E-08 |
| quantity of natural killer cells | -1.89 |  | 1.55E-06 |  |
| quantity of phagocytes | 0.62 | -0.77 | 1.75E-08 | 9.92E-07 |
| quantity of regulatory T lymphocytes | -1.54 |  | 1.44E-05 |  |
| quantity of T lymphocytes | -3.17 | -1.33 | 8.95E-19 | 5.36E-08 |
| recruitment of leukocytes | -3.25 | 1.40 | 7.55E-06 | 4.08E-07 |
| response of lymphocytes | -0.85 |  | 2.20E-09 |  |
| response of macrophages | -0.33 |  | 7.70E-07 |  |
| response of phagocytes | -0.07 |  | 3.44E-06 |  |
| T cell development | -2.71 | -0.17 | 1.79E-17 | 1.20E-05 |
| T cell homeostasis | -2.93 |  | 1.95E-17 |  |
| T cell migration | -2.99 |  | 2.02E-10 |  |
| T cell response | -0.82 |  | 3.35E-07 |  |
