# Supplementary material for: The effect of age on the intestinal mucus thickness, microbiota composition and immunity in relation to sex in mice
Source: PLoS One. 2017 Sep 12;12(9):e0184274. doi: 10.1371/journal.pone.0184274 (PMC5595324; doi:10.1371/journal.pone.0184274)
Supplement: S6 Table — A p-value of <0.05 was used. The z-score gives an indication of the activation or inhibition of the functions in old versus young mice. (DOCX) [file pone.0184274.s012.docx]

**S6 Table.** Selection of immunological functions that are related to the genes with a different expression in young (3 months) and old (19 months) mice in both male and female B6 mice in the proximal colon. A p-value of <0.05 was used. The z-score gives an indication of the activation or inhibition of the functions in old versus young mice.

| **Diseases or Functions Annotation** | **z-score**  **MO vs MY** | **z-score**  **FO vs FY** | **p-Value**  **MO vs MY** | **p-Value**  **FO vs FY** |
| --- | --- | --- | --- | --- |
| activation of antigen presenting cells | -0.070 | 1.503 | 1.55E-06 | 1.45E-05 |
| activation of B lymphocytes | -1.697 |  | 2.05E-05 |  |
| activation of leukocytes | 0.378 | 1.260 | 6.38E-14 | 4.75E-11 |
| activation of lymphocytes | -0.472 | -0.050 | 4.55E-08 | 2.32E-06 |
| activation of macrophages | 0.548 |  | 1.31E-06 |  |
| activation of phagocytes | 0.827 | 1.905 | 1.91E-10 | 1.09E-07 |
| activation of T lymphocytes | -0.302 | 0.405 | 1.21E-06 | 3.00E-06 |
| adhesion of immune cells | -0.139 |  | 6.55E-10 |  |
| Bacterial Infections | 0.276 | -0.067 | 8.60E-09 | 3.91E-06 |
| cell proliferation of T lymphocytes | -0.296 | 1.143 | 1.95E-07 | 1.12E-05 |
| development of leukocytes | -0.849 |  | 1.53E-06 |  |
| development of lymphocytes | -0.575 |  | 7.21E-06 |  |
| differentiation of B lymphocytes | -0.292 |  | 3.80E-05 |  |
| differentiation of leukocytes | 1.476 |  | 3.29E-09 |  |
| differentiation of lymphocytes | -0.065 |  | 2.04E-06 |  |
| function of leukocytes | -1.480 | 0.292 | 1.25E-13 | 4.27E-08 |
| function of phagocytes | -1.982 |  | 4.23E-10 |  |
| homing of leukocytes | -0.746 | 0.377 | 2.62E-06 | 4.91E-06 |
| inflammatory response | 0.924 | 1.406 | 3.54E-16 | 2.45E-08 |
| leukocyte migration | -0.940 | -1.184 | 2.75E-17 | 2.76E-12 |
| production of antibody | 0.700 |  | 2.61E-09 |  |
| proliferation of B lymphocytes | 0.064 |  | 3.58E-09 |  |
| proliferation of immune cells | 0.280 | -0.126 | 4.61E-15 | 2.78E-07 |
| proliferation of lymphocytes | -0.021 | 0.058 | 8.60E-14 | 1.64E-06 |
| quantity of antigen presenting cells | -0.176 | -0.835 | 2.05E-10 | 2.78E-09 |
| quantity of B lymphocytes | -1.713 |  | 9.17E-11 |  |
| quantity of eosinophils | -0.080 |  | 1.62E-05 |  |
| quantity of granulocytes | 0.873 |  | 2.63E-07 |  |
| quantity of leukocytes | -0.632 | -1.064 | 1.73E-23 | 2.62E-14 |
| quantity of lymphocytes | -1.479 | -2.148 | 1.91E-17 | 1.61E-09 |
| quantity of macrophages | 0.684 | -0.765 | 7.03E-10 | 1.57E-07 |
| quantity of neutrophils | 1.302 |  | 6.79E-07 |  |
| quantity of phagocytes | 0.844 | 0.350 | 7.67E-12 | 8.93E-11 |
| quantity of plasma cells | 0.904 |  | 2.46E-05 |  |
| quantity of T lymphocytes | -0.387 | -1.499 | 3.25E-10 | 1.09E-05 |
| recruitment of antigen presenting cells | -0.747 |  | 6.24E-06 |  |
| recruitment of granulocytes | -1.146 | -1.229 | 2.52E-07 | 2.92E-07 |
| recruitment of leukocytes | -1.268 | -0.675 | 8.33E-11 | 4.60E-09 |
| recruitment of macrophages | -0.722 |  | 6.11E-06 |  |
| recruitment of neutrophils | -1.338 | -1.065 | 5.28E-06 | 1.25E-05 |
| recruitment of phagocytes | -1.159 |  | 5.56E-08 |  |
